# Synthesis, Molecular Structure and Spectroscopic Investigations of Novel Fluorinated Spiro Heterocycles

**DOI:** 10.3390/molecules20058223

**Published:** 2015-05-07

**Authors:** Mohammad Shahidul Islam, Abdullah Mohammed Al-Majid, Assem Barakat, Saied M. Soliman, Hazem A. Ghabbour, Ching Kheng Quah, Hoong-Kun Fun

**Affiliations:** 1 Department of Chemistry, College of Science, King Saud University, P.O. Box 2455, Riyadh 11451, Saudi Arabia; E-Mail: amajid@ksu.edu.sa; 2 Department of Chemistry, Faculty of Science, Alexandria University, P.O. Box 426, Ibrahimia, Alexandria 21321, Egypt; E-Mail: saied1soliman@yahoo.com; 3 Department of Chemistry, Rabigh College of Science and Art, King Abdulaziz University, P.O. Box 344, Rabigh 21911, Saudi Arabia; 4 Department of Pharmaceutical Chemistry, College of Pharmacy, King Saud University, P.O. Box 2457, Riyadh 11451, Saudi Arabia; E-Mails: ghabbourh@yahoo.com (H.A.G.); hfun.c@ksu.edu.sa (H.-K.F.); 5 X-ray Crystallography Unit, School of Physics, Universiti Sains Malaysia, Penang 11800, Malaysia; E-Mail: ckquah@usm.my

**Keywords:** fluorine spiro-heterocycles, [5+1] cycloaddition, *N*,*N*-dimethylbarbituric acid, X-ray, DFT

## Abstract

This paper describes an efficient and regioselective method for the synthesis of novel fluorinated spiro-heterocycles in excellent yield by cascade [5+1] double Michael addition reactions. The compounds 7,11-*bis*(4-fluorophenyl)-2,4-dimethyl- 2,4-diazaspiro[5.5] undecane-1,3,5,9-tetraone (**3a**) and 2,4-dimethyl-7,11-bis (4-(trifluoromethyl)phenyl)-2,4-diazaspiro[5.5]undecane-1,3,5,9-tetraone (**3b**) were characterized by single-crystal X-ray diffraction, FT-IR and NMR techniques. The optimized geometrical parameters, infrared vibrational frequencies and NMR chemical shifts of the studied compounds have also been calculated using the density functional theory (DFT) method, using Becke-3-Lee-Yang-Parr functional and the 6-311G(d,p) basis set. There is good agreement between the experimentally determined structural parameters, vibrational frequencies and NMR chemical shifts of the studied compounds and those predicted theoretically. The calculated natural atomic charges using NBO method showed higher polarity of **3a** compared to **3b**.The calculated electronic spectra are also discussed based on the TD-DFT calculations.

## 1. Introduction

Spiro-compounds, especially heterocycles, are present in a large number of plants and animals. A wide range of natural alkaloids possessing a spiro-carbon in their molecules have proven their biological activity in agriculture and medicine. They also act as nicotinic receptor antagonists and have anticancer, antibiotic and antimicrobial properties. Additionally, a review of the literature has shown that the synthesis of fused or spiro-heterocyclic compounds derived from cycloalkanes has not been explored; however, in many cases, it has been observed that enlargement of the cycloalkane ring size influences the biological effect—cyclododecane derivatives appear to have an advantage in this regard [1,2,3,4,5,6,7,8,9].

As an example, we can highlight the fact that some diazaspiro[5.5]-undecane-1,3,5,9-tetraone motifs have a large range of therapeutic and biological properties such as anticonvulsant [10], potent sedative-hypnotic [11], CNS depressant properties [12], anti-fungicidal [13], and antibacterial activity [14].

On the other hand, the importance of fluorine in medicinal, agricultural, and material chemistry is well recognized and its role in drug design has been reviewed regularly over the years and several fluorinated compounds are currently being widely used to treat diverse diseases. It has also been highlighted that fluorine substitution can alter the chemical properties, disposition, and biological activity of many drugs and moreover, due to the special nature of fluorine atom, many properties of certain medicines, including enhanced binding interactions, metabolic stability, changes in physical properties, and selective reactivity, can be modified, and flourine inclusion can influence both the disposition of the drug and the interaction with the respective pharmacological target [15,16,17,18,19,20,21,22,23,24].

The Michael addition reaction is one of the most remarkable tools for C-C bond construction in organic synthesis [25,26,27,28,29,30,31,32]; in particular intermolecular double-Michael reactions are the most powerful tool for assembling spirocyclic products from the non-cyclic starting materials.

As a part of our ongoing program to develop efficient and robust methods for the preparation of biologically relevant compounds from readily available building blocks [25,26,27,28,29,30,31,32], we have extended our work to synthesize some new fluorine compounds derived from barbituric acid spiro-heterocycles. Herein, we report the regioselective synthesis in excellent yield of 7,11-*bis*(4-fluorophenyl)-2,4-dimethyl-2,4-diazaspiro[5.5]undecane-1,3,5,9-tetraone (**3a**) 2,4-dimethyl-7,11-*bis*(4-(trifluoromethyl)-phenyl)-2,4-diazaspiro[5.5]undecane-1,3,5,9-tetraone (**3b**). The studied compounds have been characterized using single crystal X-ray diffraction (XRD), FTIR and NMR spectroscopy. The two compounds differ in the F-substitution present on the phenyl rings. Since the substituent has peculiar characteristics influencing the electronic and spectroscopic aspects of compounds we employed density functional theory (DFT) in conjunction with the B3LYP/6-311G(d,p) to predict the electronic and spectroscopic properties (FTIR, UV-Vis and NMR) of the studied compounds.

## 2. Results and Discussion

### 2.1. Synthesis of **3a**,**b**

The synthetic pathway to the title compounds is summarized in Table 1. The starting compound, *N*,*N*-dimethylbarbituric acid (**1**) is commercially available, and (1*E*,4*E*)-1,5-*bis*(4-fluorophenyl)penta-1,4-dien-3-one (**2a**) and (1*E*,4*E*)-1,5-*bis*(4-(trifluoromethyl)phenyl)penta-1,4-dien-3-one (**2b**) were obtained by the condensation of *p*-fluorobenzaldehyde and *p*-trifluoromethylbenzaldehyde with acetone, respectively [28,29]. The reaction of **1** with equimolar amounts of the dienones **2a**,**b** in DCM using NHEt_2_ as a base afforded the target compounds 7,11-*bis*(4-fluorophenyl)-2,4-dimethyl-2,4-diazaspiro[5.5]undecane-1,3,5,9-tetraone (**3a**) and 2,4-dimethyl-7,11-*bis*(4-(trifluoro- methyl)phenyl)-2,4-diazaspiro[5.5]undecane-1,3,5,9-tetraone (**3b**) regioselectively in excellent yield. The reaction between the nucleophilic center in **1** and the bielectrophilic centers in **2a**,**b**, in principle, can yield two regioisomers, however, in the present study due to different reactivity of nucleophilic and electrophilic sites, only a single isomer was isolated. The compounds were characterized by ^1^H-, ^13^C-NMR and GCMS studies.

**Table 1:** Cascade [5+1] double Michael addition reaction as key step to form spirocyclic products **3a**,**b**. 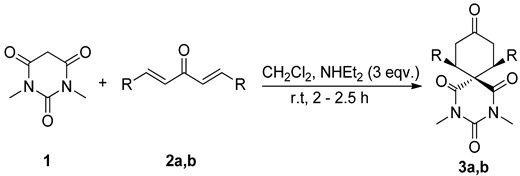

Entries ^a^	Diarylidene 2a,b	R	3	Yield (%) ^b^
1	**2a**	*p*-FPh	**3a**	98
2	**2b**	*p*-CF_3_Ph	**3b**	94

^a^ Reaction conditions: *N*,*N*-dimethylbarbituric acid (**1**, 2 mmol), diarylideneacetone derivatives **2a**,**b** (2 mmol); ^b^ isolated yield.

**Figure 1:**
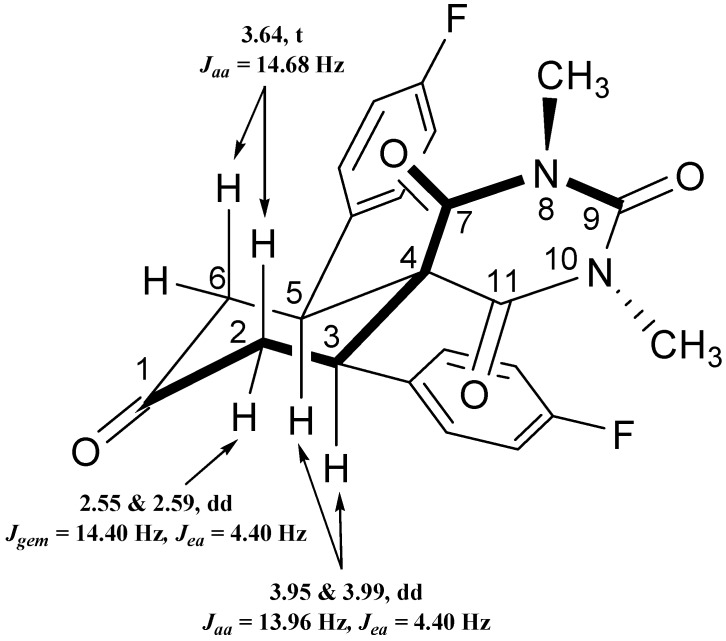
Structure representation for compound **3a**.

In the ^1^H-NMR spectra, the two N-Me protons at positions 8 and 10 of compounds **3a**,**b** are strongly deshielded because these protons are flanked by two adjacent carbonyl groups and both N-Me groups are chemically non-identical because of the anisotropy effect caused by the carbonyl groups present at the C_8_ and C_11_ positions (Figure 1).

The ^1^H-NMR spectrum of the diazaspiro compound **3a** shows a doublet of doublets at 2.55 and 2.59 with coupling constants *J*_gem_ = 15.40 Hz and *J*_ea_ = 4.4 Hz, for the equatorial protons at the C_2_ and C_6_ positions, while another doublet of doublets appeared at 3.95 and 3.99 with coupling constants *J*_aa_ = 13.96 Hz and *J*_ea_ = 4.40 Hz attributed to the axial protons at the C_3_ and C_5_ positions. On the other hand one triplet appeared at 3.64 with coupling constant *J*_aa/gem_ = 14.68 Hz for the axial protons present at the C_2_ and C_6_ positions (Figure 1). 

Similarly the ^1^H-NMR spectrum of the diazaspiro compound **3b** shows a doublet of doublets at 2.70 and 2.74 with coupling constants *J*_gem_ = 15.40 Hz and *J*_ea_ = 4.4 Hz and one triplet at 3.56 with coupling constant *J*_aa/gem_ = 14.68 Hz, representing for the equatorial and axial protons at the C_2_ and C_6_ positions, respectively, while another doublet of doublets appeared at 4.25 and 4.28 with coupling constants *J*_aa_ = 13.96 Hz and *J*_ea_ = 4.40 Hz due to the axial protons at the C_3_ and C_5_ positions. The ^19^F-NMR spectrum shows single signals at −112.36 and −62.65 for the specific F-atom and CF_3_-group respectively.

Here, the axial protons at positions C_2_, C_3_, C_4_ and C_6_ are less shielded as compared to the equatorial protons at the C_2_ and C_6_ position, because of the proximity of the phenyl ring to the C_3_ and C_4_positions, therefore the equatorial protons at positions C_2_ and C_6_ appeared at higher field (δ 2.55–2.74 ppm) than the axial protons. The anisotropic effect of C=O on Heq at C_2_ and C_6_ is also responsible for the observed diamagnetic shift of these protons.

### 2.2. X-ray Crystal Structures of **3a**,**b**

**Figure 2:**
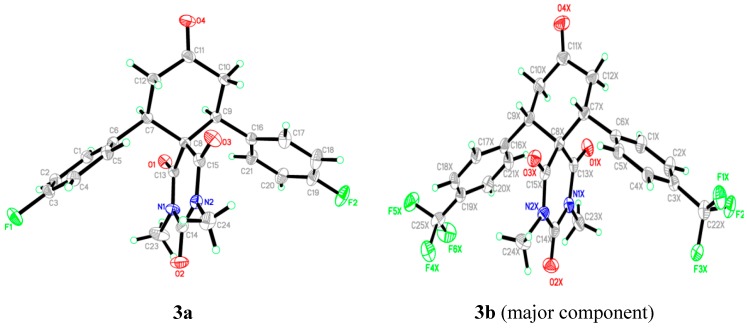
The ORTEP diagram of the final X-ray model of compounds **3a**,**b** with displacement ellipsoids drawn at 30% probability level. The minor disorder component of compound **3b** is omitted for clarity.

The compounds **3a**,**b** were obtained as single crystals by slow diffusion at room temperature for 2 days with diethyl ether of pure compounds **3a**,**b** in dichloromethane. Data was collected on a Bruker APEX-II D8 Venture area diffractometer (Bruker AXS GmbH, Karlsruhe, Germany), equipped with graphite monochromatic Mo Kα radiation at 100 (2) K. Cell refinement and data reduction was carried out by Bruker SAINT (Bruker AXS GmbH, Karlsruhe, Germany). SHELXS-97 [33,34] was used to solve these structures. The final refinement was carried out by full-matrix least-squares techniques with anisotropic thermal data for non-hydrogen atoms on 𝐹^2^. All the hydrogen atoms were placed in calculated positions (Table 2). Similarity and simulation restraints were applied for the disordered atoms. The crystal structures of **3a**,**b** are shown in Figure 2. CCDC-1034099; and CCDC-1034833 contain the supplementary crystallographic data for these compounds. The data can be obtained free of charge from the Cambridge Crystallographic Data Centre via http://www.ccdc.cam.ac.uk/data_request/cif (or from the CCDC, 12 Union Road, Cambridge CB2 1EZ, UK; Fax: +44 1223 336033; E-mail: deposit@ccdc.cam.ac.uk).

**Table 2:** The crystal and experimental data of compounds **3a** and **3b**.

Properties	Compound 3a	Compound 3b
Empirical formula	C_23_H_20_F_2_N_2_O_4_	C_25_H_20_F_6_N_2_O_4_
Formula weight	426.41	526.43
Crystal system	Monoclinic	Tetragonal
Space group	*C*2/*c*	*P-42_1_c*
Unit cell dimensions	a = 33.1745 (16)Å, α = 90.00° b = 7.7860 (3)Å β = 100.68 (3)° c = 15.866 (7)Å, γ = 90.00°	a = 22.1813 (10)Å, α = 90.00° b = 22.1813 (10) Å, β = 90.00° c = 9.2692 (4)Å, γ = 90.00°
Volume	4027.2 (3)Å^3^	4560.5 (5)Å^3^
Density (calculated)	1.407Mg/m^3^	1.533 Mg/m^3^
Absorption coefficient	0.11 mm^−1^	0.14 mm^−1^
*F*(000)	1776	2160
Crystal size	0.65 × 0.27 × 0.15 mm	0.59 × 0.33 × 0.21mm
Theta range for data collection	2.5°–30.5°.	2.4°–30.5°.
Index ranges	−47 ≤ *h* ≤ 47, −11 ≤ *k* ≤ 11, −22 ≤ *l* ≤ 22	−26 ≤ *h* ≤ 26, −26 ≤ *k* ≤ 26, −10 ≤ *l* ≤ 11
Reflections collected/ unique	112794/ 4917 [R(int) = 0.038]	35961 / 4754 [R(int) = 0.024] 82456/4010[R(int) = 0.053]
Completeness to theta = 30.57°	99.7%	99.9%
Goodness-of-fit on *F^2^*	1.03	1.09
Largest diff. peak and hole	0.54 and −0.27 e·Å^−3^	0.27 and −0.26 e·Å^−3^
CCDC number	1034099	1034833

**Figure 3:**
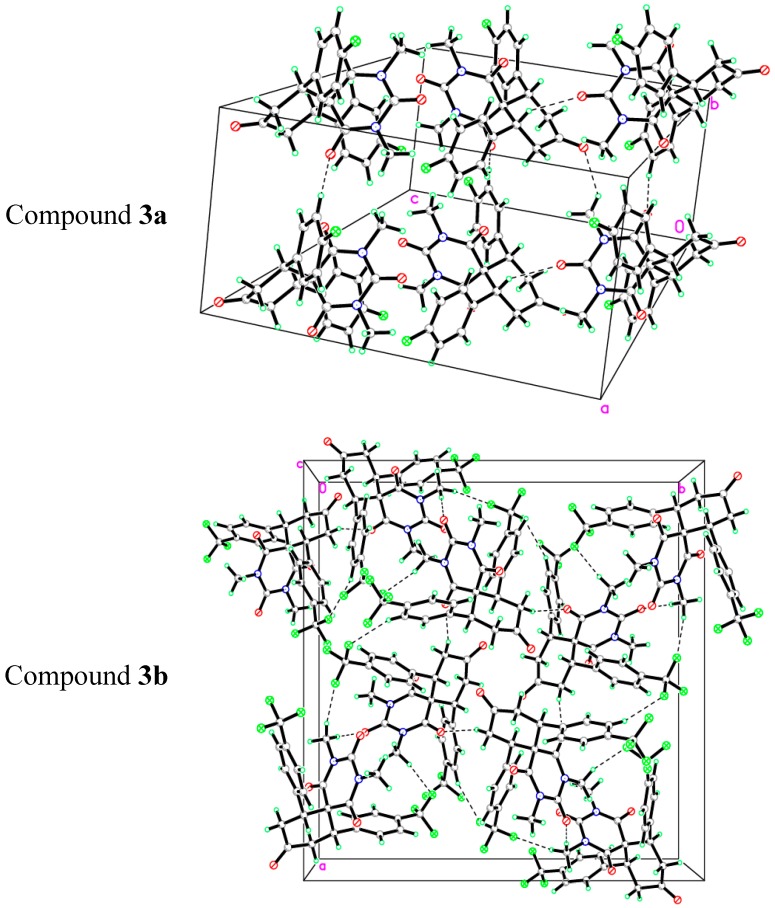
View of the molecular packing in compounds **3a**,**b**.

The asymmetric unit of compound **3a** contains one molecule (Figure 2). In the molecule, the pyrimidine rings are in chair form. Bond lengths and angles are within normal ranges. The cyclohexanone ring (C7–C12) is almost perpendicular to the mean plane of pyrimidine ring (C8/C13/N1/C14/N2/C15) with the dihedral angle of 86.76 (2)°. Also the phenyl rings (C1–C6) and (C16–C21) have dihedral angles of 72.74 (2)° and 58.59 (1)°, respectively, with the pyrimidine ring (C8/C13/N1/C14/N2/C15). In the crystal structure, Figure 3 (copound **3a**), the molecules are linked via intermolecular C2-H2A•••O3, C9-H9A•••O2, C24-H24B•••F1 and C2-H24C•••O4 hydrogen bonds (see Supporting Information). 

**Figure 4:**
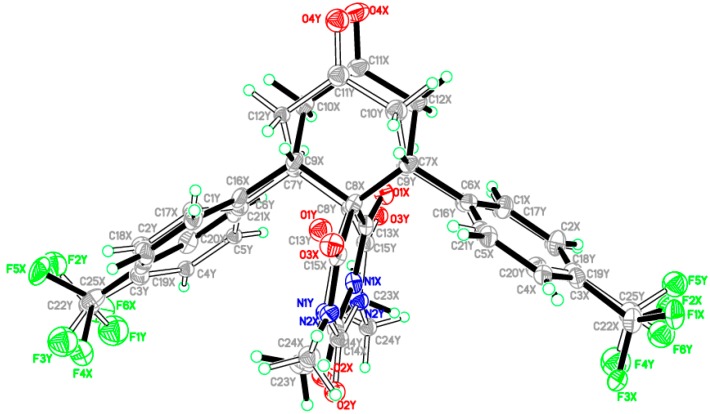
The ORTEP diagram of the final X-ray model of compound **3b** with full molecule disorder.

The asymmetric unit of compound **3b** contains one molecule (Figure 2). In this, the whole molecule ring system is disordered over two sets of sites with refined site occupancies of 0.799 (1): 0.201 (1), suggesting 180° rotational disorder for the diazaspiro[5.5]undecane-1,3,5,9-tetraone ring system. The pyrimidine rings are in chair form conformation. Bond lengths and angles are within normal ranges. In the molecule (Figure 4), the cyclohexanone ring (C7–C12) is almost perpendicular to the mean plane of pyrimidine ring (C8/C13/N1/C14/N2/C15) with the dihedral angles of 85.04 (2) and 83.21 (4) for major and minor components, respectively. In the crystal structure, Figure 3, the molecules are linked into a 3-dimensional network via intermolecular C4X-H4XA•••F5X, C10X-H10B•••O3X, C23X-H23A•••F4X, C23X-H23B•••O2X and C24X-H24A•••F3X hydrogen bonds (see Supporting Information).

### 2.3. Computational Details

All the quantum chemical calculations of the studied compounds were performed by applying DFT method with the B3LYP functional and 6-311G(d,p) basis set using Gaussian 03 software [35]. The input files were taken from the CIF obtained from our reported X‒ray single crystal measurements. The geometries were optimized by minimizing the energies with respect to all the geometrical parameters without imposing any molecular symmetry constraints. GaussView 4.1 [36] and Chemcraft [37] programs have been used to draw the structure of the optimized geometries and to visualize the HOMO and LUMO pictures. Frequency calculations at the optimized geometries were done to confirm the optimized structures to be an energy minimum. The true energy minimum at the optimized geometry of the studied compounds was confirmed by absence of any imaginary frequency modes. The electronic spectra of the studied compounds were calculated by the TD‒DFT method. The gauge including atomic orbital (GIAO) method was used for NMR calculations. The ^1^H and the ^13^C isotropic shielding tensors referenced to the TMS calculations were carried out at the same level of theory. The natural atomic charges were calculated using NBO method as implemented in the Gaussian 03 package [38] at the DFT/B3LYP level. The results of the computed geometrical parameters of compounds **3a**,**b** are reported and discussed with respect to the X-ray data. This is followed by discussion of the experimental and theoretical electronic and spectroscopic properties (IR, UV-Vis and NMR) of the studied compounds. The electronic spectra are also discussed based on the TD-DFT results.

#### 2.3.1. Optimized Molecular Geometry

**Figure 5:**
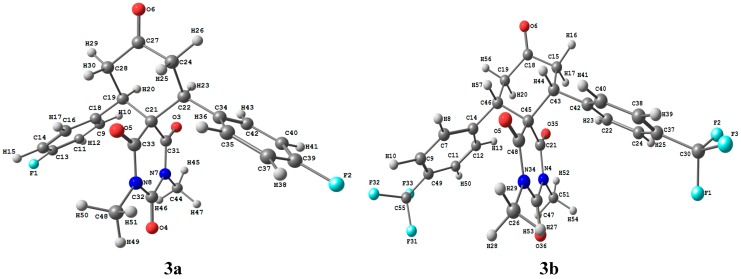
The optimized molecular structure of the studied compounds **3a**,**b**.

To clarify the electronic and spectroscopic properties of the studied compounds correctly, it is essential to examine the geometry of the studied compounds. A small change in the geometry can potentially cause substantial variations in their electronic and spectroscopic aspects. The optimized geometric structures of **3a**,**b** are shown in Figure 5. The experimental and theoretical geometrical parameters (bond lengths and bond angles) are listed in Table S7 (Supplementary Information). The maximum deviations of the calculated bond length and bond angle values from the experimental data are 0.019Å (C21‒C22) and 1.915° (C18‒C19‒C21), The root mean square deviation (RMSD), the mean absolute deviation (MAD) and the correlation coefficient (R^2^) values between the experimental and calculated bond lengths are found to be 0.009 Å–0.078 Å–0.9979 and 0.017 Å–0.077 Å–0.9891 for **3a** and **3b**, respectively. Also, the RMSD, MAD and R^2^ values between the experimental and calculated bond angles are found to be 0.806°–3.552°–0.9840 and 1.674°–4.741°–0.9562 for for **3a** and **3b**, respectively. The deviations of the calculated geometric parameters from the experimental results because the latter were obtained at a very low temperature and the molecular rotations were restricted, while the theoretical results were obtained for a single molecule in the gas phase without any intermolecular interactions. The geometric parameters are predicted very well.

Charge distribution plays a vital role in the application of quantum chemical calculations to molecular systems because atomic charges affect many properties such as dipole moment, molecular polarizability and electronic structure of compound [39]. In this regard, the natural atomic charges (NAC) were calculated using NBO method at the DFT B3LYP/6-311G(d,p) level of theory and the results are given in Table 3. The natural charge calculations indicate the electronegative nature of the F, O and N-atoms. The maximum natural charges occur at the *O*-atoms are in the range −0.5416 to −0.6260 and −0.5377 to −0.6309 for **3a** and **3b**, respectively. The dipole moments of **3a** and **3b** are calculated to be 2.3021D and 0.9163D, respectively. Interestingly, **3a**, which has F-substituent directly attached to the phenyl rings, is more polar than **3b**, which has a CF_3_ substituent bonded to the phenyl rings. Since the skeleton of the spiro structure which has the electron rich oxygen atoms is common in both compounds so we could predict the polarity difference between the two molecules due to difference of the substituent at phenyl rings. In **3a**, the *F*-atoms present at the *para*-position of the phenyl groups result in an electron withdrawing effect on these phenyl rings and may thus lead to a molecular system with electron withdrawing units at both ends of the molecule. It appears that this effect is smal in the **3b** molecule and so a low dipole moment has been predicted.

**Table 3:** The natural atomic charges calculated at the B3LYP/6-311G (d,p) method.

3a				3b			
F1	−0.3468	H29	0.2283	F1	−0.3625	H29	0.2228
F2	−0.3468	H30	0.2368	F2	−0.3629	C30	1.1118
O3	−0.6191	C31	0.7398	F3	−0.3634	F31	−0.3620
O4	−0.5981	C32	0.8567	N4	−0.4888	F32	−0.3634
O5	−0.6260	C33	0.7240	O5	−0.6182	F33	−0.3637
O6	−0.5416	C34	−0.0596	O6	−0.5377	N34	−0.4900
N7	−0.4909	C35	−0.1934	C7	−0.1889	O35	−0.6309
N8	−0.4889	H36	0.2154	H8	0.2176	O36	−0.5886
C9	−0.1784	C37	−0.2606	C9	−0.1661	C37	−0.1514
H10	0.2162	H38	0.2212	H10	0.2209	C38	−0.1676
C11	−0.2624	C39	0.4327	C11	−0.1643	H39	0.2222
H12	0.2221	C40	−0.2624	C12	−0.2038	C40	−0.1892
C13	0.4327	H41	0.2221	H13	0.2178	H41	0.2177
C14	−0.2606	C42	−0.1784	C14	−0.0235	C42	−0.0237
H15	0.2212	H43	0.2162	C15	−0.4814	C43	−0.1975
C16	−0.1934	C44	−0.3530	H16	0.2295	H44	0.2294
H17	0.2154	H45	0.2226	H17	0.2381	C45	−0.1819
C18	−0.0596	H46	0.2051	C18	0.6236	C46	−0.1975
C19	−0.1937	H47	0.2052	C19	−0.4813	C47	0.8539
H20	0.2275	C48	−0.3535	H20	0.2382	C48	0.7362
C21	−0.1845	H49	0.2266	C21	0.7232	C49	−0.1515
C22	−0.1937	H50	0.2021	C22	−0.2037	H50	0.2192
H23	0.2275	H51	0.2021	H23	0.2179	C51	−0.3552
C24	−0.4814			C24	−0.1625	H52	0.2223
H25	0.2368			H25	0.2174	H53	0.2068
H26	0.2283			C26	−0.3536	H54	0.2069
C27	0.6238			H27	0.2061	C55	1.1118
C28	−0.4814			H28	0.2068	H56	0.2294

#### 2.3.2. Infrared Vibrational Studies

**Figure 6:**
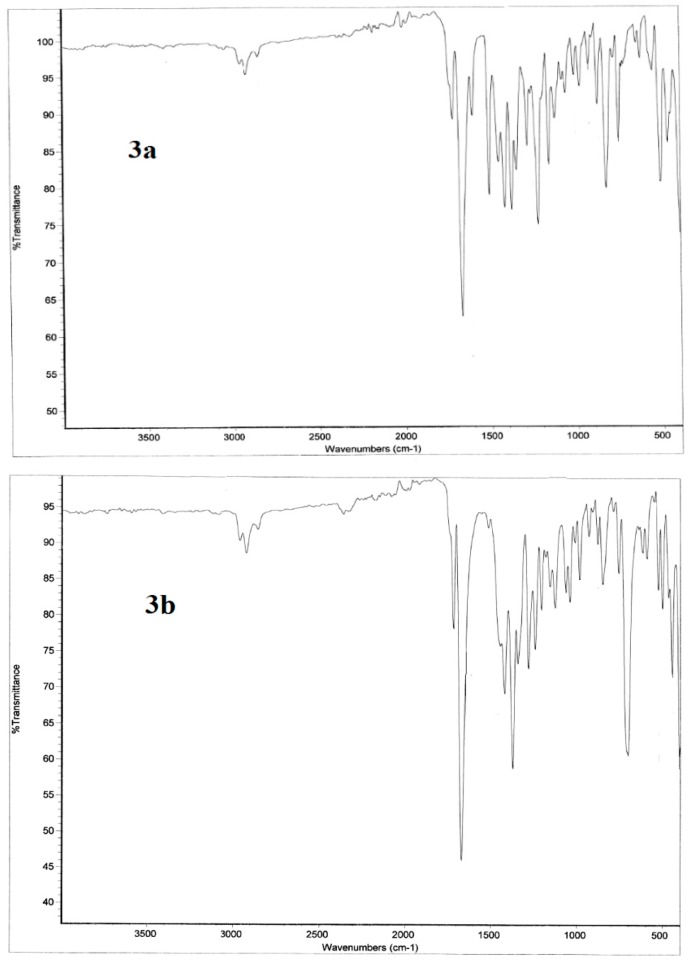
The experimental infrared vibrational spectra of the studied compounds **3a**,**b**.

**Figure 7:**
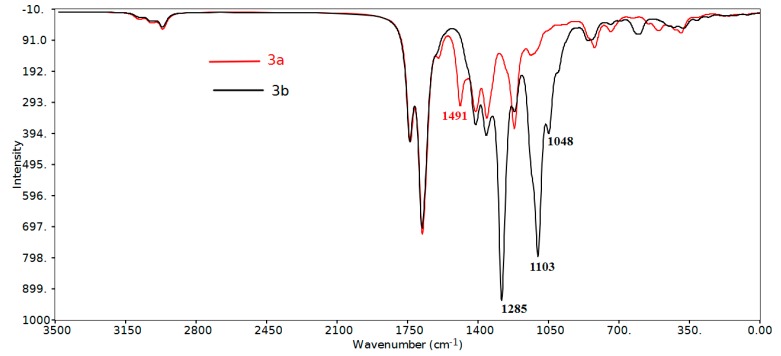
The calculated scaled [44] infrared vibrational spectra of the studied compounds.

The studied compounds belong to C_1_ point group. The compounds **3a**,**b** consist of 51 and 57 atoms, having 147 and 165 normal vibrational modes, respectively. The assignments of the vibrational fundamental modes for the investigated compounds are given in Table 4. The experimental and simulated vibrational spectra are depicted in Figure 6 and Figure 7, respectively. The correlation coefficients between the calculated and experimental IR vibrational frequencies are 0.9994 and 0.9997 for **3a** and **3b** respectively. We noted that, the IR spectral bands in the region above 1650 cm^−1^ are almost the same for the two studied compounds. The region below 1650 cm^−1^ showed significant changes due to the variation of the F-substitution at the phenyl rings. The following are some of the important vibrational motions observed.

The C-H stretching vibrations of aromatic structures generally occur in the region 3100–3000 cm^−1^ [40] while the saturated hydrocarbon shows C-H stretching absorption bands in the region 3000–2840 cm^−1^ [41]. Both molecules have two types of C-H bonds: eight aromatic and 12 aliphatic C-H bonds. The aromatic stretching bands of the **3a** and **3b** molecules are calculated at 3096, 3095, 3080 and 3073 cm^−1^ (exp. 2956 cm^−1^) and 3101–3093, 3082–3074 cm^−1^ (exp. 2956 cm^−1^), respectively. For the aliphatic C-H bonds, the asymmetric C-H stretching vibrations of **3a** and **3b** are calculated at 3087, 3084, 3033–3007 cm^−1^ (exp. 2921 cm^−1^) and 3085, 3033–3008 cm^−1^ (exp. 2919 cm^−1^). The symmetric C-H modes usually appear at lower frequencies and higher intensity of 2970–2952 cm^−1^ (exp. 2850 cm^−1^) and 2971–2954 cm^−1^ (exp. 2851 cm^−1^) for the **3a** and **3b**, respectively. Moreover, the four carbonyl vibrations predicted at 1740–1668 cm^−1^ (exp. 1718–1688 cm^−1^) and 1743–1661 cm^−1^ (1715–1669 cm^−1^) for the **3a** and **3b**, respectively.

##### Region below 1650 cm^−1^

The asymmetric and symmetric bending vibrations of methyl groups normally appear in the 1470–1440 cm^−1^ and 1390–1370 cm^−1^ region, respectively [42,43]. These bending modes are predicted theoretically and observed experimentally in their characteristic regions (Table 4). The CH_3_ umbrella vibrations are calculated at 1422 and 1403–1355 cm^−1^ (exp. 1378, 1349 cm^−1^) and 1423 and 1400–1355 cm^−1^ (exp. 1373, 1343 cm^−1^) while the CH_2_ rocking vibrations are calculated at 1416–1404 cm^−1^ (exp. 1418 cm^−1^) and 1416–1405 cm^−1^ (exp. 1419 cm^−1^) for **3a** and **3b**, respectively.

**Table 4:** The calculated and experimental wavenumbers of the studied compounds **3a**,**b**.

Assignment	3a		3b	
	Calculated	Experimental	Calculated	Experimental
υ_(CH, aromatic)_	3096, 3095, 3080, 3073	2956	3101–3093, 3082–3074	2956
υ_(CHasym, aliphatic)_	3087, 3084, 3033–3007	2921	3085, 3033–3008	2919
υ_(CHsym, aliphatic)_	2970–2952	2850	2971–2954	2851
υ_(C=O)_	1740–1668	1718-1688	1743–1661	1715–1669
υ_C=C (aromatic)_	1595–1491	1604, 1508	1604–1497	1512
δ_CH asym methyl_	1460–1449	1454	1463–1448	1448
δ_CH sym methyl_	1422, 1403–1355	1378, 1349	1423, 1400–1355	1373, 1343
δ_CH rocking CH2_	1416–1404	1418	1416-1405	1419
δ_CH aromatic in plane_^a^	1492, 1491, 1281–998	1508, 1286–980	1497, 1300–1020	1512, 1281–1008
υ_(C-CF3)_			1285, 1284	1281
υ_(C-F)_	1223–1219	1224	1103, 1048 (asym) 1000 (sym)	1038 (asym) 1008 (sym)
δ_CH aromatic out-of-plane_	948–806	928–784	965–823	980–847

υ: streching δ: bending; ^a^: mixed with other vibrations.

##### Region above 1650 cm^−1^

The aromatic ring C-H in-plane bending vibrations are usually weak and observed in the 1300–998 cm^−1^ region (exp. 1286–980 cm^−1^), while the C-H out-of-plane bending vibrations lie in the 965–806 cm^−1^ region (exp. 980–784 cm^−1^). We noted a high frequency C-H in-plane bending vibrations at 1497–1492 cm^−1^ (exp. 1512–1508 cm^−1^) mixed with the aromatic C=C stretching modes. Theoretically, this band was predicted to have higher intensity in **3a** compared to **3b**. Usually the aromatic C-F stretching vibrations appeared in the overlapping region with the aromatic C-H in plane vibrations. In the **3a** molecule, the υ_C-F_ modes are predicted at 1223–1219 cm^−1^ (exp. 1224 cm^−1^). We noted the C-F in-plane and out-of-plane bending modes at 423 and 308 cm^−1^, respectively. Like the methyl group, the CF_3_ groups of the **3b** molecule have two asymmetric and one symmetric stretching modes. The former is calculated at 1103 and 1048 cm^−1^ (exp. 1038 cm^−1^) while the latter at 1000 cm^−1^ (exp. 1008 cm^−1^). Moreover a strong intense C-C(CF_3_) stretching vibration at ~1285 cm^−1^ (exp. 1281 cm^−1^) has been predicted for compound **3b**.

#### 2.3.3. Frontier Molecular Orbitals

**Figure 8:**
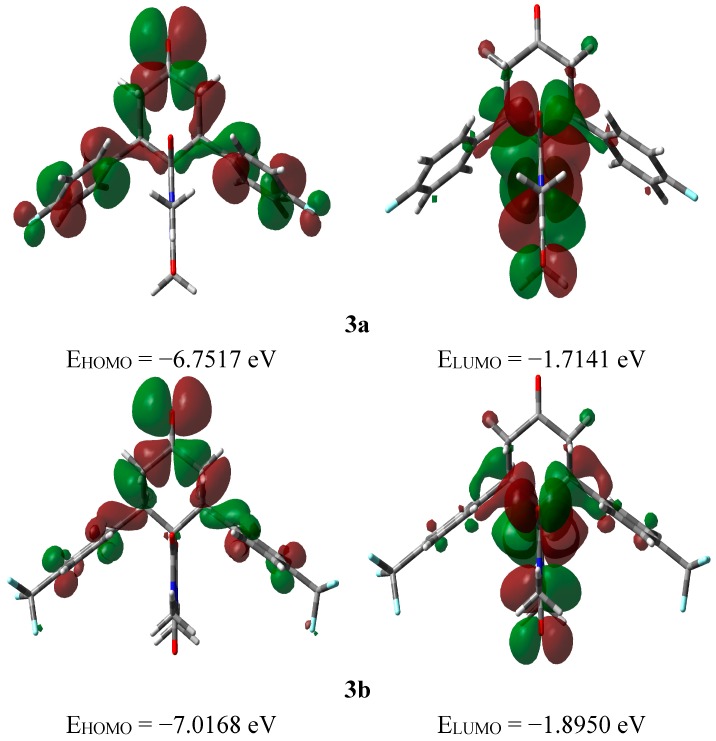
The ground state isodensity surface plots for the frontier molecular orbitals.

**Figure 9:**
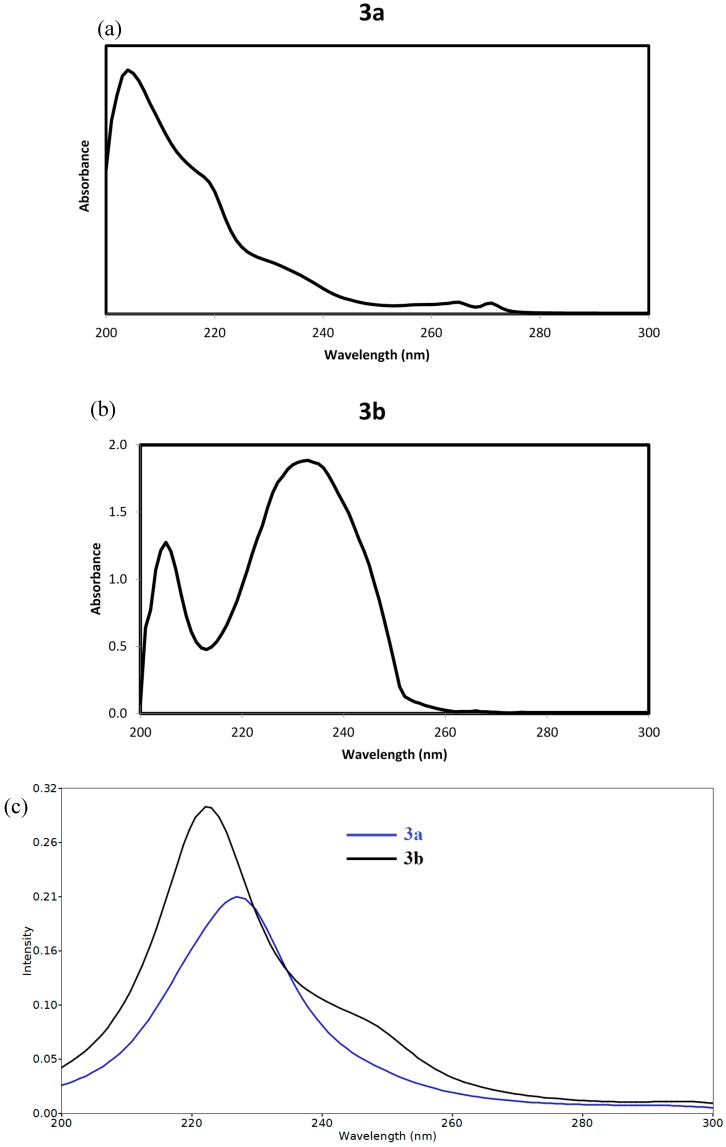
(**a**) The experimental electronic spectra of the studied compounds **3a**; (**b**) The experimental electronic spectra of the studied compounds **3b**; (**c**) The calculated electronic spectra of the studied compounds using TD-DFT method.

The electron densities of the frontier molecular orbitals (FMOs) were used for predicting the most reactive position in π-electron systems and also explained several types of reactions in conjugated system [45]. The energy values of the lowest unoccupied molecular orbital (E_LUMO_) and the highest occupied molecular orbital (E_HOMO_) and their energy gap (ΔE) reflect the chemical reactivity of the molecule. Recently, the energy gap between HOMO and LUMO has been used to prove the bioactivity from intramolecular charge transfer (ICT) [46,47]. The E_HOMO_, E_LUMO_ and ΔE values of the studied compound were calculated by B3LYP/6‒311G(d,p) method and the HOMO and LUMO pictures are shown in Figure 8. The electron densities of the HOMO and LUMO are mainly localized on the π-system of the studied molecules. It is found that the E_HOMO_ of **3a** and **3b** are −6.7517 and −7.0168 eV, respectively while the E_LUMO_ values are −1.7141 and −1.8950 eV, respectively. The **3a** molecule has lower energy gap (5.0377 eV) than the **3b** one (5.1218 eV). The presence of F-atom directly attached to the phenyl ring enhances the ICT interactions. The time-dependant density functional theory (TD-DFT) enabled us to accurately describe the possible electronic transitions of the molecular systems. In this regard, the twenty spin allowed singlet-singlet electronic transitions calculated using TD-DFT results were collected in Table S8 (Supplementary Information). The experimental and calculated electronic spectra of **3a** and **3b** are shown in Figure 9.

Compound **3a** showed an intense absorption band calculated at 228.3 nm (f = 0.1530) assigned to the H − 1/H→L + 2 and H→L + 3/L + 5 transitions. This band observed experimentally as a shoulder at 233 nm in dichloromethane. Compound **3b** showed an intense transition band at 222.1 nm (f = 0.2486) and a shoulder at 248.5 nm (f = 0.0363) due to the H − 1→L + 1 and H→L + 1/L + 5 transitions, respectively. Experimentally, the longest wavelength band was observed at 232nm.

#### 2.3.4. NMR Spectra

The isotropic magnetic shielding (IMS) values calculated using the GIAO approach at the 6-311G(d,p) level were used to predict the ^13^C and ^1^H chemical shifts (δ_calc_) for the studied compounds and the results are correlated with the experimental NMR data (δ_exp_) in CDCl_3_ solvent. The experimental and theoretical ^1^H- and ^13^C-NMR chemical shift values of the studied compounds are given in Table S3 (Supplementary Information). According to these results, the calculated chemical shifts comply with the experimental findings. The correlations between experimental and the calculated chemical shifts are very good (Figures S1 and S2, Supplementary Materials). The correlation coefficients (R^2^) are calculated to be in the range 0.949–0.989. Generally, the aromatic carbons give signals in overlapped areas of the spectrum with chemical shift values from 100 to 150 ppm [48,49]. Based on the ^13^C-NMR spectra, the presence of a heteroatom such as fluorine attached to the carbon will shift the peak downfield (higher chemical shift). Since C13 and C39 of **3a** are attached to the F-atoms, a high chemical shift is expected for these C-atoms (δ_calc_ = 172.4 ppm). On other hand, the aliphatic C-atoms usually give signals below 50 ppm. The C30 and C55 have high chemical shift values (~134.24 ppm) because each of these C-atoms is attached to three F-atoms, which have high electronegativity.

## 3. Experimental Section

### 3.1. General

All glassware was oven-dried before use and the reactions were conducted under an inert atmosphere. The progress of the reaction was monitored by TLC (Silica Gel 60 F–254 thin layer plates, Merck, Schwalbach; Hessen, Germany). The chemicals were purchased from Aldrich (Gillingham, Dorset, UK) and Fluka Chemie GmbH (Buchs, Switzerland), *etc.*, and were used without further purification, unless otherwise stated. Petroleum ether (PE), hexane and ethyl acetate were distilled prior to use especially for column chromatography. All the major solvents were dried by using standard drying techniques mentioned in the literature. Melting points were measured on a Gallenkamp melting point apparatus in open glass capillaries and are uncorrected. IR Spectra were measured as KBr pellets on a 6700 FT-IR Nicolet spectrophotometer (Thermo Fisher Scientific, Madison, WI, USA). The NMR spectra were recorded on a Jeol-400 NMR spectrometer (Peabody, MA, USA). 1H-NMR (400 MHz), and 13C-NMR (100 MHz) were recorded in deuterated chloroform (CDCl_3_). Chemical shifts (δ) are referred in terms of ppm and J-coupling constants are given in Hz. Mass spectrometric analysis was conducted by using ESI mode on an Agilent Technologies 6410–triple quad LC/MS instrument (Santa Clara, CA, USA). Elemental analysis was carried out on a Perkin Elmer 2400 Elemental Analyzer, CHN mode (Waltham, MA, USA). The electronic spectra of the studied compounds 3a and 3b were measured using a Perkin Elmer Lambda 35, UV/Vis spectrophotometer (Waltham, MA, USA). 

### 3.2. General Procedure of Double Michael Addition Reaction for the Synthesis of Spiro-Compounds **3a**,**b** (**GP1**)

A solution of *N*,*N*-dimethylbarbituric acid (**1**, 2 mmol) and diarylidene acetone derivatives **2a**,**b** (2 mmol) in dry CH_2_Cl_2_ (10 mL) was charged into a 50 mL round bottom flask under inert atmosphere. Et_2_NH (2.5 mmol) was then added to the reaction mixture which was stirred at room temperature for up to 1.5–2 h, until TLC showed complete consumption of both the reactants. After the completion of reaction, the crude product was directly subjected to column chromatography using 100–200 mesh silica gel and ethyl acetate/*n*-hexane (2:8, v/v) as an eluent to afford the pure products **3a**,**b**. The solid products were further crystallized from a mixture of CHCl_3_/*n*-heptane.

#### 3.2.1. 7,11-bis(4-Fluorophenyl)-2,4-dimethyl-2,4-diazaspiro[5.5]undecane-1,3,5,9-tetraone (3a)

Diarylideneacetone **2a** (540 mg, 2 mmol) was reacted with compound **1** (312.1 mg, 2 mmol) according to GP1 to yield a white solid of spiro-product **3a** (835 mg, 1.96 mmol, 98%); m.p. 175–177 °C; UV-Vis: 204 nm, 218 nm (sh) and 233 nm (sh) (in Dichloromethane); ^1^H-NMR (CDCl_3_) δ: 2.55 and 2.59 (dd, 2H, *J*_gem_ = 15.40 Hz, *J*_ea_ = 4.40 Hz, C**H**_2(e)_), 2.88 (s, 3H, -NC**H**_3_), 3.02 (s, 3H, –NC**H**_3_), 3.64 (t, 2H, *J*_aa_ = 14.68 Hz, C**H_2(a)_**), 3.95 & 3.99 (dd, 2H, *J*_aa_ = 13.96 Hz, *J*_ae_ = 4.40 Hz, C**H**), 6.89–6.94 (m, 4H, Ar-**H**), 7.01–7.04 (m, 4H, Ar-**H**): ^13^C-NMR (CDCl_3_) δ: 27.92, 28.34, 42.93, 49.56, 60.82, 115.73 and 115.95 (d, *J*^2^ = 21.4 Hz), 115.10 and 129.18 (d, *J*^3^ = 7.65 Hz), 132.78 and 132.81 (d, *J*^4^ = 3.06 Hz), 149.33, 161.20 & 163.67 (d, *J*^1^ = 147 Hz), [ArC_1_, C_2_, C_3_& C_4_ are split into doublets due to ^19^F], 168.74, 170.48, 207.27; IR (KBr, cm^−1^) ν_max_ = 2920, 1718, 1618, 1507, 1418, 1378, 1223, 1159, 828, 753, 510, 466; [Anal. Calcd. for C_23_H_20_F_2_N_2_O_4_: C, 50.39; H, 3.68; N, 5.11; Found: C, 50.51; H, 3.73; N, 5.17]; LC/MS (ESI, *m/z*): [M^+^], found 426.21, C_23_H_20_F_2_N_2_O_4_ requires 426.14.

#### 3.2.2. 2,4-Dimethyl-7,11-bis (4-(trifluoromethyl)phenyl)-2,4-diazaspiro[5.5]undecane-1,3,5,9-tetraone (3b)

Diarylideneacetone **2b** (684 mg, 2 mmol) was reacted with compound **1** (312.1 mg, 2 mmol) according to GP1 to yield the white solid spiro-product **3b** (936 mg, 1.88 mmol, 94%); m p. 180–182 °C; UV-Vis: (in ethanol): 204nm and 232nm; ^1^H-NMR (CDCl_3_) δ: 2.70 and 2.74 (dd, 2H, *J*_gem_ = 15.40 Hz, *J*_ea_ = 4.40 Hz, C**H**_2(e)_), 3.02 (s, 3H, -NC**H**_3_), 3.04 (s, 3H, –NC**H**_3_), 3.56 (t, 2H, *J*_aa_ = 14.68 Hz, C**H**_2(e)_), 4.25 and 4.28 (dd, 2H, *J*aa = 13.96 Hz, *J*ae = 4.40 Hz, C**H**), 6.75–7.14 (m, 4H, Ar-**H**); ^13^C-NMR (CDCl_3_) δ: 28.21, 28.63, 44.17, 45.44, 61.51, 125.48, 125.87, 126.96, 139.84, 149.93, 168.60, 170.91, 205.78; IR (KBr, cm^−1^) ν_max_ = 2956, 2919, 1715, 1669, 1419, 1373, 1280, 702, 500, 444; [Anal. Calcd. for C_23_H_16_F_6_N_2_O_4_: C, 55.43; H, 3.24; N, 5.62; Found: C, 55.29; H, 3.17; N, 5.75]; LC/MS (ESI, *m/z*): [M^+^], found 498.19, C_23_H_16_F_6_N_2_O_4_ requires 498.10.

## 4. Conclusions

In conclusion, we have synthesized novel spiro-heterocyclic molecules by room temperature cascade [5+1] double Michael addition reactions using diethylamine as a strong base. The molecular structures of the studied compounds have been optimized using the DFT/B3LYP method and 6-311G(d,p) basis set. The calculated bond distances and bond angles showed good agreement with our reported X-ray crystal structures. The IR vibrational frequencies are calculated and the fundamental bands were assigned and compared with the experimental data. The regions above 1650 cm^−1^ have common spectral patterns in both compounds but this is not the case for the region below this value. The aromatic υ_C-F_ modes are predicted at 1223–1219 cm^−1^ (exp. 1224 cm^−1^) while the asymmetric and symmetric C-F stretching modes are calculated at 1,103 and 1048 cm^−1^ (exp. 1038 cm^−1^) and 1000 cm^−1^ (exp. 1008 cm^−1^), respectively. The natural atomic charges at the different atomic sites calculated using NBO method were used to describe the higher polarity of **3a** compared to **3b**.The calculated electronic spectra using the TD-DFT showed the most intense absorption band at 228.3 nm (f = 0.1530) for **3a** and 222.1 nm (f = 0.2486) for **3b**. The GIAO ^1^H- and ^13^C-NMR chemical shift values correlated well with the experimental data (R^2^ = 0.949–0.989). The F-atoms has significant effect on the chemical shifts of the C-atoms attached to them.

## Supplementary Materials

Supplementary materials can be accessed at: http://www.mdpi.com/1420-3049/20/05/8223/s1.
