# Supplementary material for: Synthesis, Molecular Structure and Spectroscopic Investigations of Novel Fluorinated Spiro Heterocycles
Source: Molecules. 2015 May 7;20(5):8223–41. doi: 10.3390/molecules20058223 (PMC6272187; doi:10.3390/molecules20058223)
Supplement: Supplementary file 1 [file molecules-20-08223-s001.pdf]

# Supplementary Materials

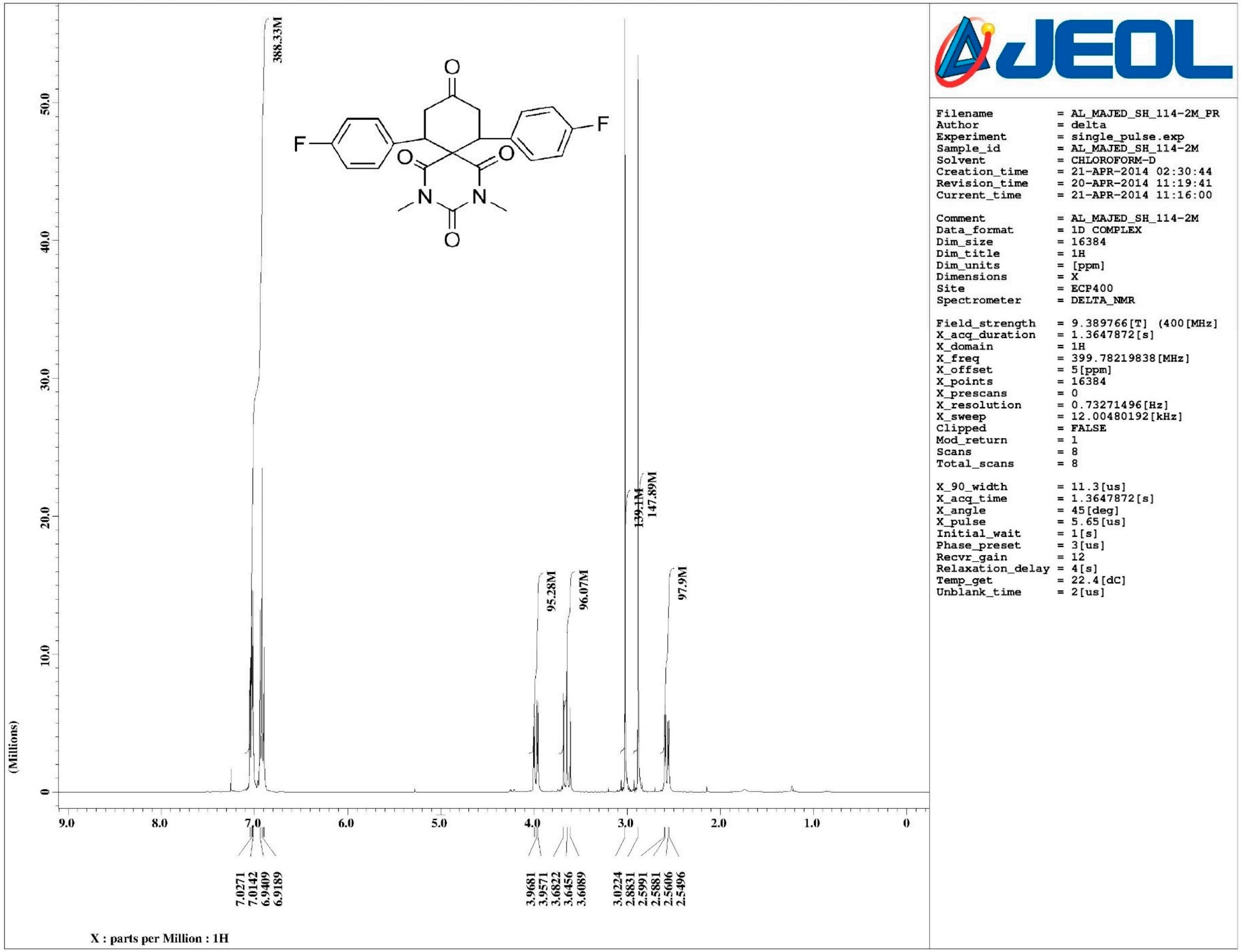


**Figure S1.** $^{1}$H-NMR spectrum of compound **3a**.

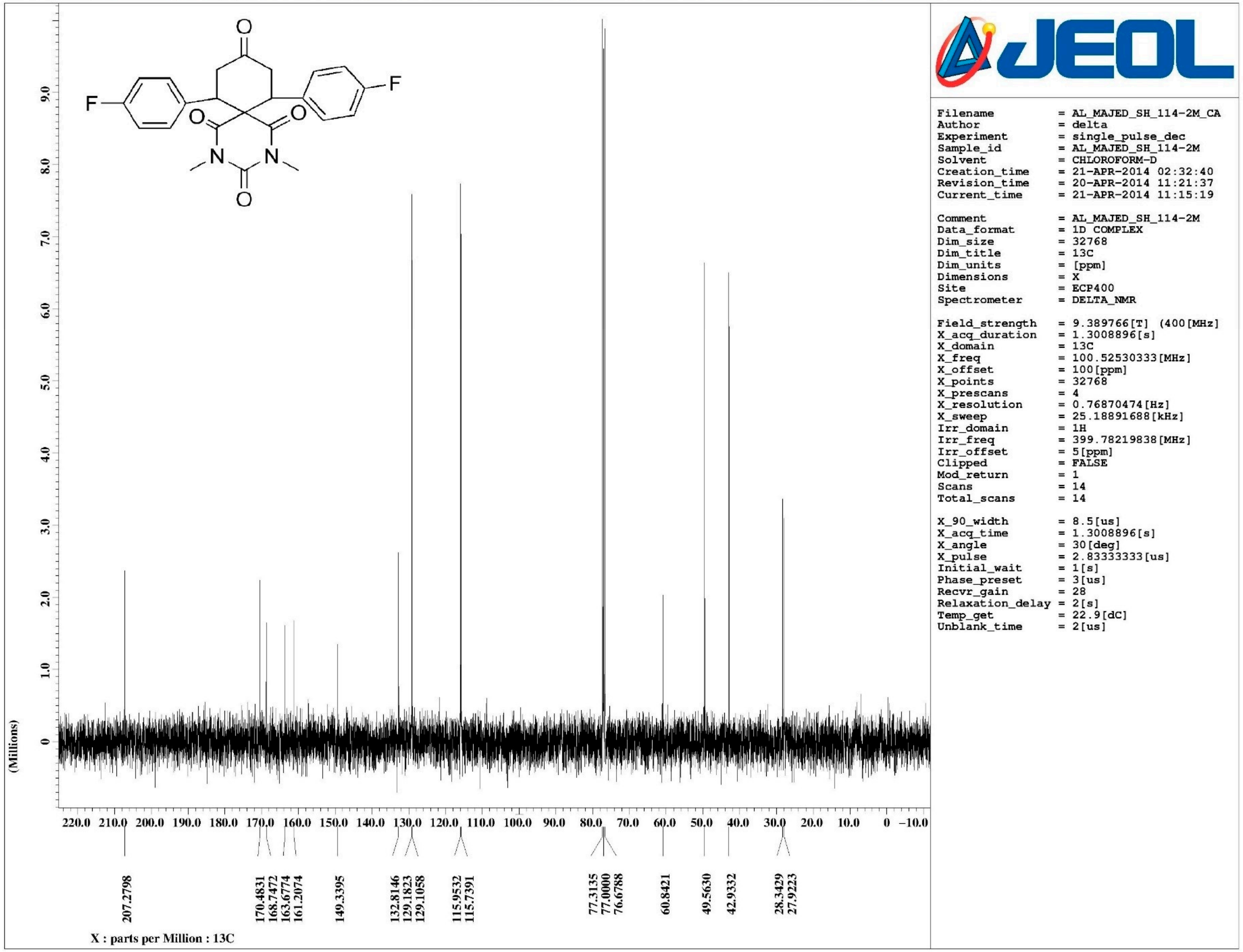


**Figure S2.** $^{13}$C-NMR spectrum of compound **3a**.

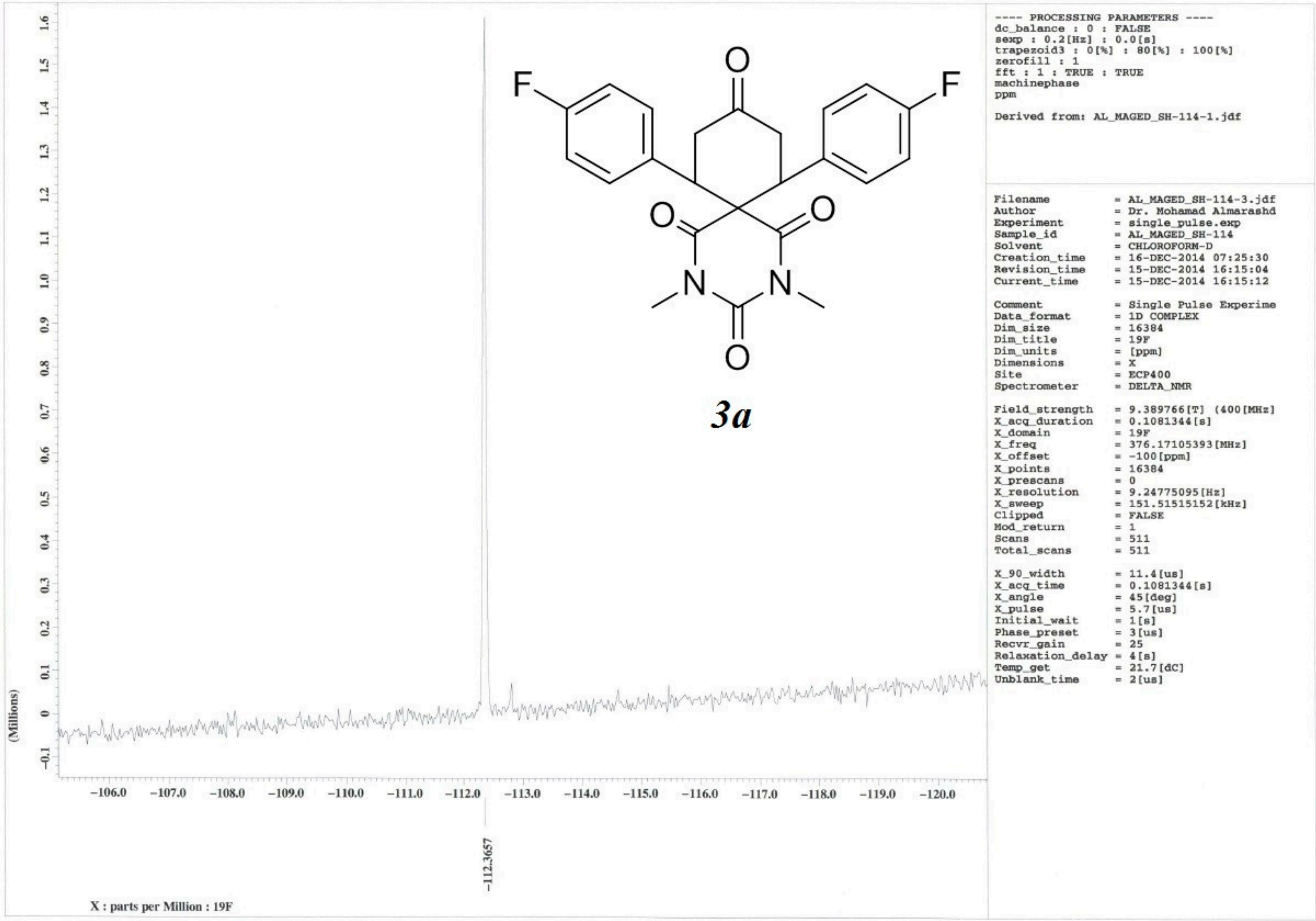


**Figure S3.** $^{19}$F-NMR spectrum of compound **3a**.

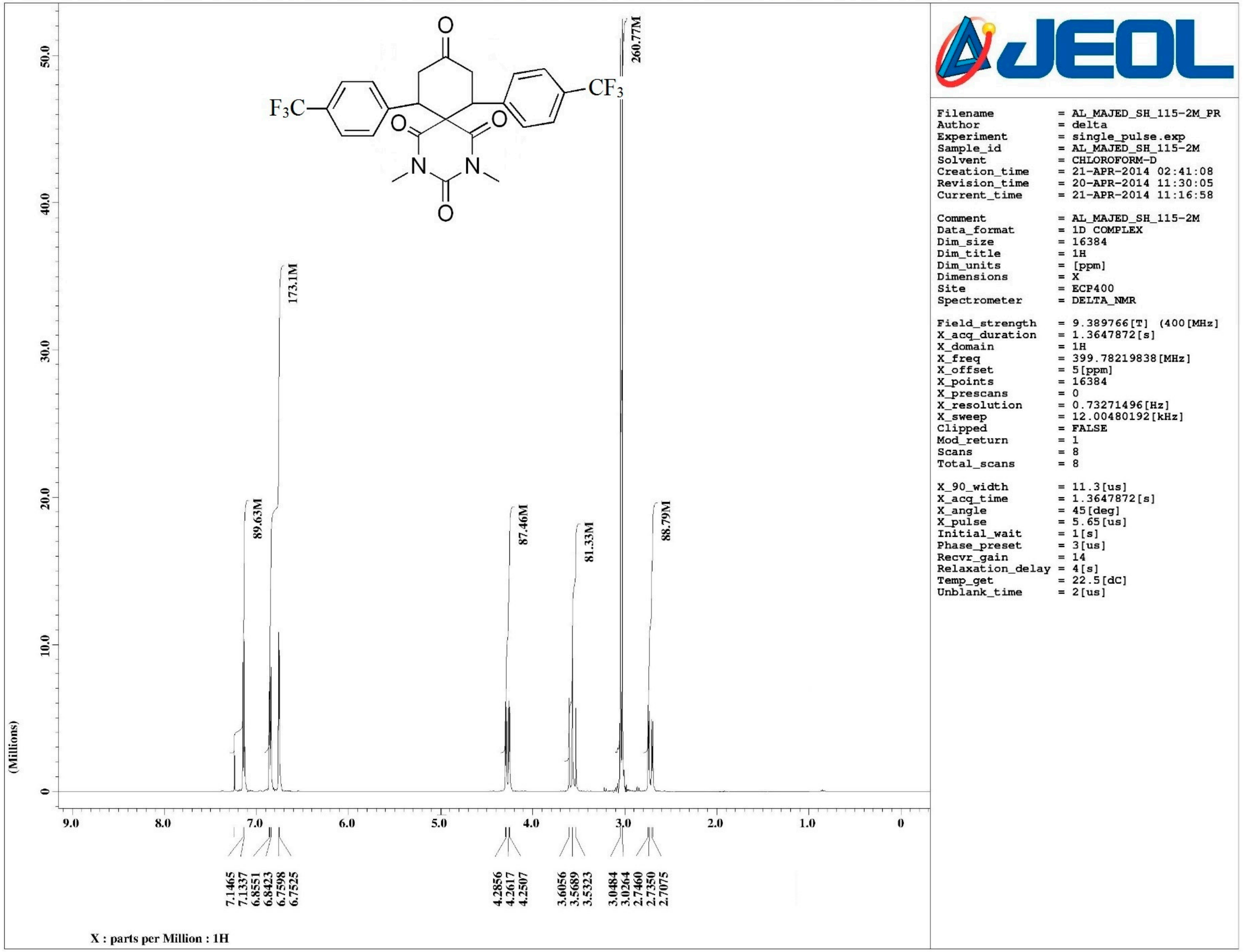


**Figure S4.** $^{1}$H-NMR spectrum of compound **3b**.

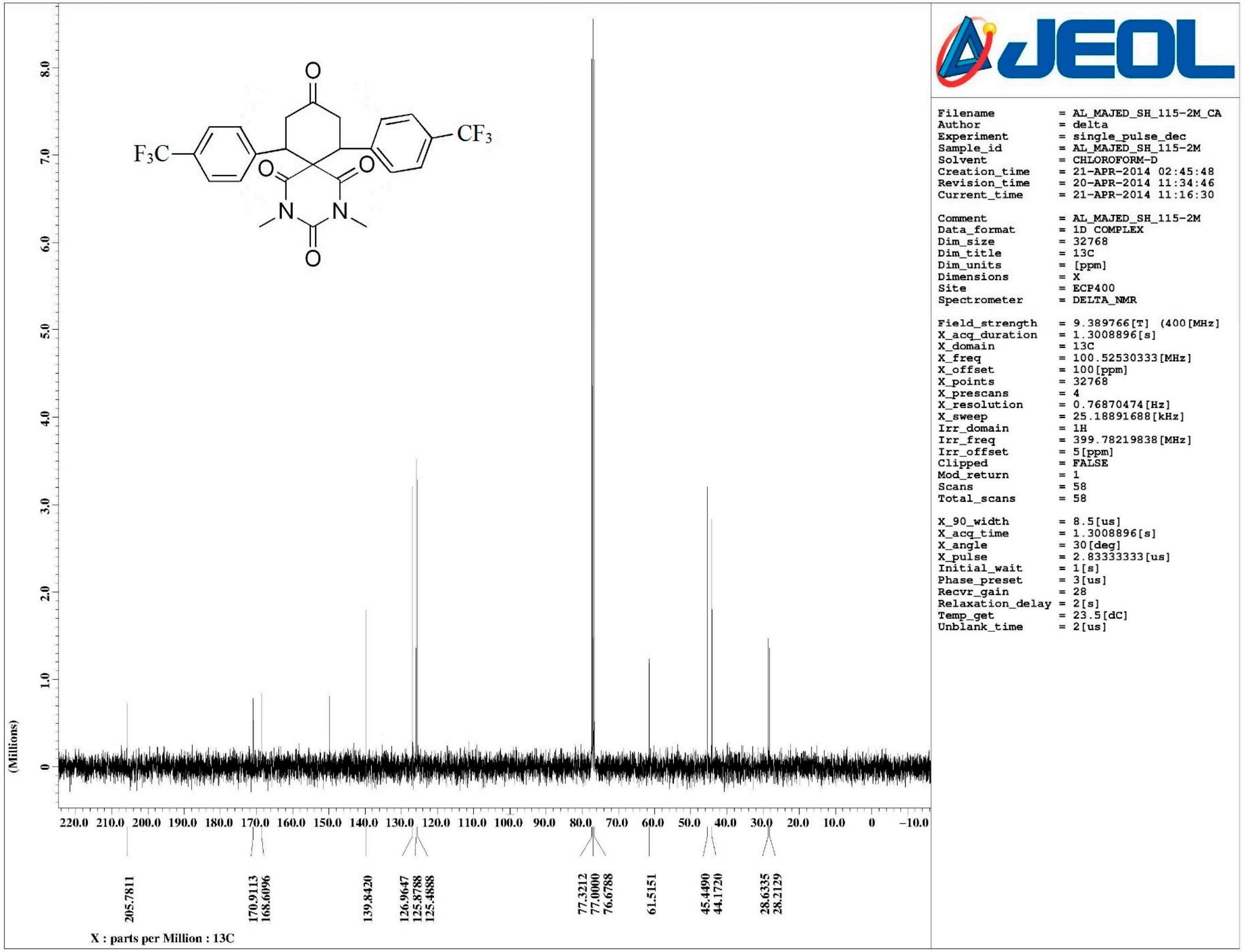

**Figure S5.** $^{13}$C-NMR spectrum of compound **3b**.

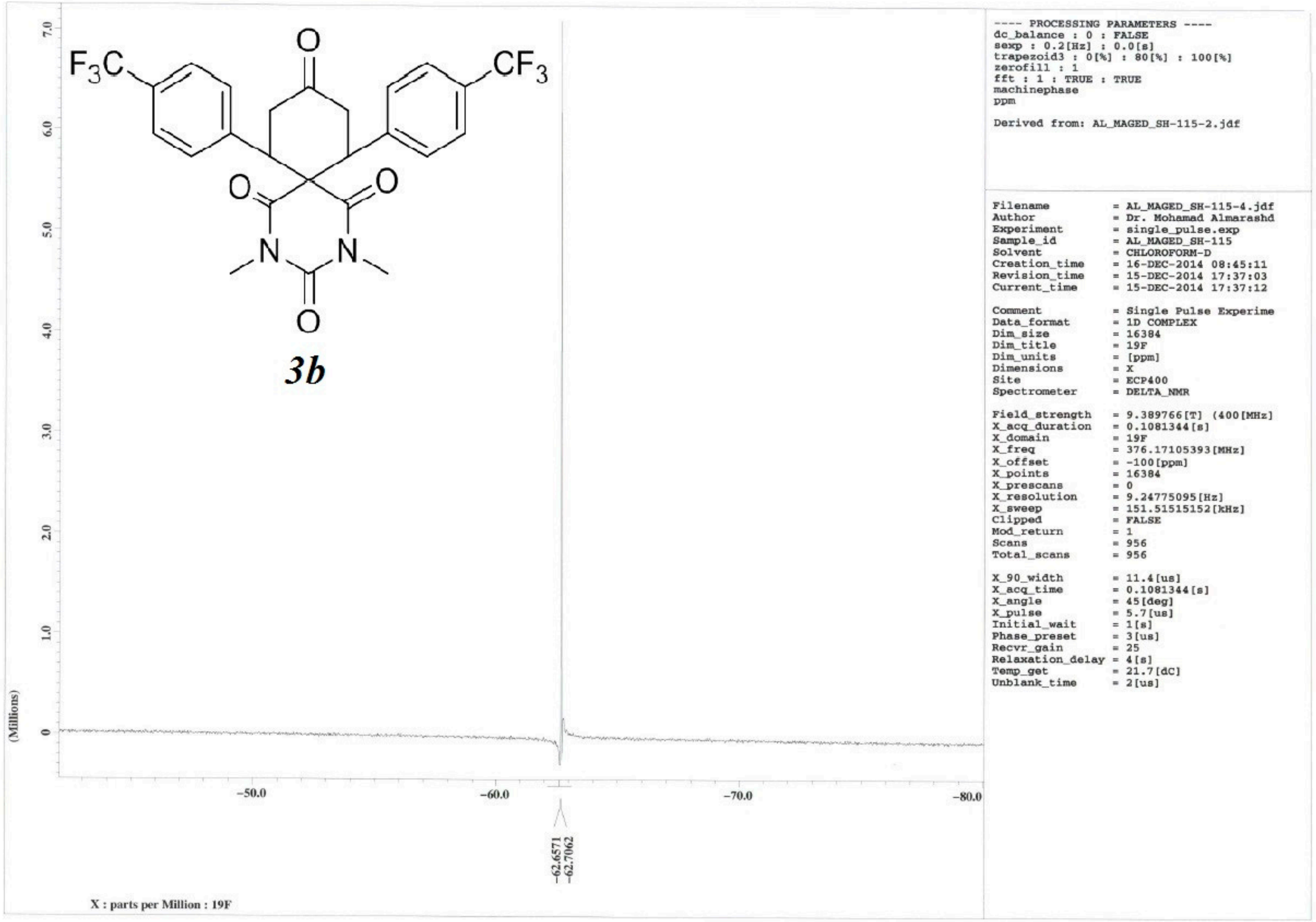


**Figure S6.** $^{19}$F-NMR spectrum of compound **3b**.

**Table S1.** Geometric parameters (Å, °) of **3a**.

| | | | |
|---|---|---|---|
| F1-C3 | 1.3604 (15) | C8-C13 | 1.5192 (17) |
| F2-C19 | 1.3656 (17) | C8-C9 | 1.5666 (17) |
| O1-C13 | 1.2167 (16) | C9-C16 | 1.5256 (18) |
| O2-C14 | 1.2099 (17) | C9-C10 | 1.5333 (18) |
| O3-C15 | 1.2176 (16) | C9-H9A | 0.9800 |
| O4-C11 | 1.2098 (17) | C10-C11 | 1.506 (2) |
| N1-C13 | 1.3770 (16) | C10-H10A | 0.9700 |
| N1-C14 | 1.3898 (17) | C10-H10B | 0.9700 |
| N1-C23 | 1.4691 (18) | C11-C12 | 1.5076 (19) |
| N2-C15 | 1.3793 (17) | C12-H12A | 0.9700 |
| N2-C14 | 1.3856 (18) | C12-H12B | 0.9700 |
| N2-C24 | 1.4710 (17) | C16-C21 | 1.3922 (19) |
| C1-C2 | 1.3835 (19) | C16-C17 | 1.393 (2) |
| C1-C6 | 1.3937 (19) | C17-C18 | 1.391 (2) |
| C1-H1A | 0.9300 | C17-H17A | 0.9300 |
| C2-C3 | 1.3763 (19) | C18-C19 | 1.370 (3) |
| C2-H2A | 0.9300 | C18-H18A | 0.9300 |
| C3-C4 | 1.376 (2) | C19-C20 | 1.374 (2) |
| C4-C5 | 1.3940 (19) | C20-C21 | 1.395 (2) |
| C4-H4A | 0.9300 | C20-H20A | 0.9300 |
| C5-C6 | 1.3967 (18) | C21-H21A | 0.9300 |
| C5-H5A | 0.9300 | C23-H23A | 0.9600 |
| C6-C7 | 1.5210 (17) | C23-H23B | 0.9600 |
| C7-C12 | 1.5311 (18) | C23-H23C | 0.9600 |
| C7-C8 | 1.5799 (18) | C24-H24A | 0.9600 |
| C7-H7A | 0.9800 | C24-H24B | 0.9600 |
| C8-C15 | 1.5134 (18) | C24-H24C | 0.9600 |
| C13-N1-C14 | 125.26 (11) | O4-C11-C10 | 121.76 (13) |
| C13-N1-C23 | 118.08 (12) | O4-C11-C12 | 121.27 (13) |
| C14-N1-C23 | 116.56 (12) | C10-C11-C12 | 116.79 (12) |
| C15-N2-C14 | 124.96 (11) | C11-C12-C7 | 113.55 (11) |
| C15-N2-C24 | 117.62 (12) | C11-C12-H12A | 108.9 |
| C14-N2-C24 | 117.34 (12) | C7-C12-H12A | 108.9 |
| C2-C1-C6 | 121.19 (12) | C11-C12-H12B | 108.9 |
| C2-C1-H1A | 119.4 | C7-C12-H12B | 108.9 |
| C6-C1-H1A | 119.4 | H12A-C12-H12B | 107.7 |
| C3-C2-C1 | 118.23 (12) | O1-C13-N1 | 120.43 (12) |
| C3-C2-H2A | 120.9 | O1-C13-C8 | 120.88 (11) |
| C1-C2-H2A | 120.9 | N1-C13-C8 | 118.65 (11) |
| F1-C3-C2 | 118.33 (12) | O2-C14-N2 | 121.67 (13) |
| F1-C3-C4 | 118.43 (12) | O2-C14-N1 | 121.09 (13) |
| C2-C3-C4 | 123.24 (12) | N2-C14-N1 | 117.23 (11) |
| C3-C4-C5 | 117.53 (12) | O3-C15-N2 | 120.23 (12) |

**Table S1.** *Cont.*

| | | | |
|---|---|---|---|
| C3-C4-H4A | 121.2 | O3-C15-C8 | 120.68 (12) |
| C5-C4-H4A | 121.2 | N2-C15-C8 | 119.04 (11) |
| C4-C5-C6 | 121.32 (12) | C21-C16-C17 | 118.13 (13) |
| C4-C5-H5A | 119.3 | C21-C16-C9 | 119.20 (12) |
| C6-C5-H5A | 119.3 | C17-C16-C9 | 122.65 (12) |
| C1-C6-C5 | 118.48 (12) | C18-C17-C16 | 121.03 (15) |
| C1-C6-C7 | 119.08 (12) | C18-C17-H17A | 119.5 |
| C5-C6-C7 | 122.41 (12) | C16-C17-H17A | 119.5 |
| C6-C7-C12 | 112.60 (11) | C19-C18-C17 | 118.61 (15) |
| C6-C7-C8 | 111.27 (10) | C19-C18-H18A | 120.7 |
| C12-C7-C8 | 112.71 (11) | C17-C18-H18A | 120.7 |
| C6-C7-H7A | 106.6 | F2-C19-C18 | 118.73 (15) |
| C12-C7-H7A | 106.6 | F2-C19-C20 | 118.49 (15) |
| C8-C7-H7A | 106.6 | C18-C19-C20 | 122.77 (14) |
| C15-C8-C13 | 114.46 (10) | C19-C20-C21 | 117.77 (15) |
| C15-C8-C9 | 111.40 (10) | C19-C20-H20A | 121.1 |
| C13-C8-C9 | 107.57 (10) | C21-C20-H20A | 121.1 |
| C15-C8-C7 | 109.94 (10) | C16-C21-C20 | 121.66 (14) |
| C13-C8-C7 | 104.36 (10) | C16-C21-H21A | 119.2 |
| C9-C8-C7 | 108.76 (10) | C20-C21-H21A | 119.2 |
| C16-C9-C10 | 112.42 (11) | N1-C23-H23A | 109.5 |
| C16-C9-C8 | 112.26 (10) | N1-C23-H23B | 109.5 |
| C10-C9-C8 | 112.78 (10) | H23A-C23-H23B | 109.5 |
| C16-C9-H9A | 106.3 | N1-C23-H23C | 109.5 |
| C10-C9-H9A | 106.3 | H23A-C23-H23C | 109.5 |
| C8-C9-H9A | 106.3 | H23B-C23-H23C | 109.5 |
| C11-C10-C9 | 114.33 (11) | N2-C24-H24A | 109.5 |
| C11-C10-H10A | 108.7 | N2-C24-H24B | 109.5 |
| C9-C10-H10A | 108.7 | H24A-C24-H24B | 109.5 |
| C11-C10-H10B | 108.7 | N2-C24-H24C | 109.5 |
| C9-C10-H10B | 108.7 | H24A-C24-H24C | 109.5 |
| H10A-C10-H10B | 107.6 | H24B-C24-H24C | 109.5 |
| C6-C1-C2-C3 | −0.1 (2) | C15-C8-C13-O1 | −176.12 (12) |
| C1-C2-C3-F1 | 179.47 (12) | C9-C8-C13-O1 | −51.76 (16) |
| C1-C2-C3-C4 | −0.2 (2) | C7-C8-C13-O1 | 63.66 (15) |
| F1-C3-C4-C5 | −179.25 (12) | C15-C8-C13-N1 | 5.85 (17) |
| C2-C3-C4-C5 | 0.4 (2) | C9-C8-C13-N1 | 130.22 (12) |
| C3-C4-C5-C6 | −0.3 (2) | C7-C8-C13-N1 | −114.37 (12) |
| C2-C1-C6-C5 | 0.2 (2) | C15-N2-C14-O2 | −178.48 (13) |
| C2-C1-C6-C7 | 178.24 (12) | C24-N2-C14-O2 | −1.8 (2) |
| C4-C5-C6-C1 | 0.0 (2) | C15-N2-C14-N1 | 2.42 (19) |
| C4-C5-C6-C7 | −177.95 (12) | C24-N2-C14-N1 | 179.10 (12) |
| C1-C6-C7-C12 | 134.22 (13) | C13-N1-C14-O2 | 177.61 (13) |

**Table S1.** *Cont.*

| | | | |
|---|---|---|---|
| C5-C6-C7-C12 | −47.84 (17) | C23-N1-C14-O2 | 1.5 (2) |
| C1-C6-C7-C8 | −98.13 (14) | C13-N1-C14-N2 | −3.3 (2) |
| C5-C6-C7-C8 | 79.81 (15) | C23-N1-C14-N2 | −179.42 (13) |
| C6-C7-C8-C15 | −60.41 (13) | C14-N2-C15-O3 | −179.81 (13) |
| C12-C7-C8-C15 | 67.19 (14) | C24-N2-C15-O3 | 3.52 (19) |
| C6-C7-C8-C13 | 62.79 (13) | C14-N2-C15-C8 | 2.71 (19) |
| C12-C7-C8-C13 | −169.61 (11) | C24-N2-C15-C8 | −173.97 (12) |
| C6-C7-C8-C9 | 177.37 (10) | C13-C8-C15-O3 | 175.90 (12) |
| C12-C7-C8-C9 | −55.03 (14) | C9-C8-C15-O3 | 53.60 (16) |
| C15-C8-C9-C16 | 60.96 (14) | C7-C8-C15-O3 | −67.04 (15) |
| C13-C8-C9-C16 | −65.24 (13) | C13-C8-C15-N2 | −6.62 (17) |
| C7-C8-C9-C16 | −177.71 (10) | C9-C8-C15-N2 | −128.92 (12) |
| C15-C8-C9-C10 | −67.26 (14) | C7-C8-C15-N2 | 110.44 (13) |
| C13-C8-C9-C10 | 166.54 (11) | C10-C9-C16-C21 | −145.41 (13) |
| C7-C8-C9-C10 | 54.07 (14) | C8-C9-C16-C21 | 86.18 (15) |
| C16-C9-C10-C11 | −176.88 (11) | C10-C9-C16-C17 | 32.76 (17) |
| C8-C9-C10-C11 | −48.74 (15) | C8-C9-C16-C17 | −95.65 (15) |
| C9-C10-C11-O4 | −141.89 (15) | C21-C16-C17-C18 | −1.3 (2) |
| C9-C10-C11-C12 | 43.01 (17) | C9-C16-C17-C18 | −179.44 (14) |
| O4-C11-C12-C7 | 141.24 (15) | C16-C17-C18-C19 | −0.1 (3) |
| C10-C11-C12-C7 | −43.63 (18) | C17-C18-C19-F2 | −179.47 (15) |
| C6-C7-C12-C11 | 176.94 (12) | C17-C18-C19-C20 | 1.2 (3) |
| C8-C7-C12-C11 | 50.05 (16) | F2-C19-C20-C21 | 179.75 (15) |
| C14-N1-C13-O1 | −179.11 (13) | C18-C19-C20-C21 | −0.9 (3) |
| C23-N1-C13-O1 | −3.0 (2) | C17-C16-C21-C20 | 1.5 (2) |
| C14-N1-C13-C8 | −1.1 (2) | C9-C16-C21-C20 | 179.80 (14) |
| C23-N1-C13-C8 | 175.00 (13) | C19-C20-C21-C16 | −0.5 (2) |

**Table S2.** Geometric parameters (Å, °) of **3b** (full molecule disorder).

| | | | |
|---|---|---|---|
| F1X-C22X | 1.343 (7) | F1Y-C22Y | 1.330 (15) |
| F2X-C22X | 1.344 (7) | F2Y-C22Y | 1.318 (16) |
| F3X-C22X | 1.311 (8) | F3Y-C22Y | 1.278 (15) |
| F4X-C25X | 1.346 (8) | F4Y-C25Y | 1.345 (16) |
| F5X-C25X | 1.371 (8) | F5Y-C25Y | 1.381 (15) |
| F6X-C25X | 1.340 (7) | F6Y-C25Y | 1.325 (15) |
| N1X-C13X | 1.384 (8) | N1Y-C14Y | 1.385 (16) |
| N1X-C14X | 1.385 (9) | N1Y-C13Y | 1.393 (15) |
| N1X-C23X | 1.468 (7) | N1Y-C23Y | 1.469 (16) |
| N2X-C15X | 1.350 (8) | N2Y-C15Y | 1.345 (14) |
| N2X-C14X | 1.377 (9) | N2Y-C14Y | 1.377 (15) |
| N2X-C24X | 1.499 (9) | N2Y-C24Y | 1.504 (15) |
| O1X-C13X | 1.223 (6) | O1Y-C13Y | 1.222 (15) |
| O2X-C14X | 1.217 (7) | O2Y-C14Y | 1.214 (13) |
| O3X-C15X | 1.210 (7) | O3Y-C15Y | 1.224 (14) |
| O4X-C11X | 1.200 (6) | O4Y-C11Y | 1.198 (13) |

**Table S2.** *Cont.*

| | | | |
|---|---|---|---|
| C1X-C6X | 1.381 (7) | C1Y-C2Y | 1.3900 |
| C1X-C2X | 1.403 (6) | C1Y-C6Y | 1.3900 |
| C1X-H1XA | 0.9300 | C1Y-H1YA | 0.9300 |
| C2X-C3X | 1.394 (7) | C2Y-C3Y | 1.3900 |
| C2X-H2XA | 0.9300 | C2Y-H2YA | 0.9300 |
| C3X-C4X | 1.374 (7) | C3Y-C4Y | 1.3900 |
| C3X-C22X | 1.474 (8) | C3Y-C22Y | 1.454 (14) |
| C4X-C5X | 1.382 (6) | C4Y-C5Y | 1.3900 |
| C4X-H4XA | 0.9300 | C4Y-H4YA | 0.9300 |
| C5X-C6X | 1.396 (7) | C5Y-C6Y | 1.3900 |
| C5X-H5XA | 0.9300 | C5Y-H5YA | 0.9300 |
| C6X-C7X | 1.532 (6) | C6Y-C7Y | 1.515 (11) |
| C7X-C12X | 1.543 (7) | C7Y-C12Y | 1.548 (13) |
| C7X-C8X | 1.552 (7) | C7Y-C8Y | 1.560 (14) |
| C7X-H7XA | 0.9800 | C7Y-H7YA | 0.9800 |
| C8X-C13X | 1.510 (7) | C8Y-C13Y | 1.512 (14) |
| C8X-C15X | 1.539 (8) | C8Y-C15Y | 1.542 (13) |
| C8X-C9X | 1.590 (7) | C8Y-C9Y | 1.573 (13) |
| C9X-C16X | 1.525 (7) | C9Y-C10Y | 1.520 (14) |
| C9X-C10X | 1.529 (7) | C9Y-C16Y | 1.525 (12) |
| C9X-H9XA | 0.9800 | C9Y-H9YA | 0.9800 |
| C10X-C11X | 1.495 (7) | C10Y-C11Y | 1.504 (14) |
| C10X-H10A | 0.9700 | C10Y-H10C | 0.9700 |
| C10X-H10B | 0.9700 | C10Y-H10D | 0.9700 |
| C11X-C12X | 1.498 (7) | C11Y-C12Y | 1.499 (13) |
| C12X-H12A | 0.9700 | C12Y-H12C | 0.9700 |
| C12X-H12B | 0.9700 | C12Y-H12D | 0.9700 |
| C16X-C21X | 1.381 (7) | C16Y-C17Y | 1.3900 |
| C16X-C17X | 1.387 (7) | C16Y-C21Y | 1.3900 |
| C17X-C18X | 1.379 (7) | C17Y-C18Y | 1.3900 |
| C17X-H17A | 0.9300 | C17Y-H17B | 0.9300 |
| C18X-C19X | 1.383 (7) | C18Y-C19Y | 1.3900 |
| C18X-H18A | 0.9300 | C18Y-H18B | 0.9300 |
| C19X-C20X | 1.390 (6) | C19Y-C20Y | 1.3900 |
| C19X-C25X | 1.489 (9) | C19Y-C25Y | 1.496 (14) |
| C20X-C21X | 1.377 (7) | C20Y-C21Y | 1.3900 |
| C20X-H20A | 0.9300 | C20Y-H20B | 0.9300 |
| C21X-H21A | 0.9300 | C21Y-H21B | 0.9300 |
| C23X-H23A | 0.9600 | C23Y-H23D | 0.9600 |
| C23X-H23B | 0.9600 | C23Y-H23E | 0.9600 |
| C23X-H23C | 0.9600 | C23Y-H23F | 0.9600 |
| C24X-H24A | 0.9600 | C24Y-H24E | 0.9600 |
| C24X-H24B | 0.9600 | C24Y-H24F | 0.9600 |
| C24X-H24C | 0.9600 | C24Y-H24G | 0.9600 |

**Table S3.** Bond Angles.

| 3a | | 3b | |
|---|---|---|---|
| C13X-N1X-C14X | 124.3 (5) | C14Y-N1Y-C13Y | 124.3 (18) |
| C13X-N1X-C23X | 118.5 (5) | C14Y-N1Y-C23Y | 117 (2) |
| C14X-N1X-C23X | 117.1 (5) | C13Y-N1Y-C23Y | 117 (2) |
| C15X-N2X-C14X | 126.1 (6) | C15Y-N2Y-C14Y | 126.5 (14) |
| C15X-N2X-C24X | 116.2 (6) | C15Y-N2Y-C24Y | 116.3 (14) |
| C14X-N2X-C24X | 117.2 (7) | C14Y-N2Y-C24Y | 116.9 (14) |
| C6X-C1X-C2X | 120.9 (5) | C2Y-C1Y-C6Y | 120.0 |
| C6X-C1X-H1XA | 119.6 | C2Y-C1Y-H1YA | 120.0 |
| C2X-C1X-H1XA | 119.6 | C6Y-C1Y-H1YA | 120.0 |
| C3X-C2X-C1X | 120.0 (5) | C1Y-C2Y-C3Y | 120.0 |
| C3X-C2X-H2XA | 120.0 | C1Y-C2Y-H2YA | 120.0 |
| C1X-C2X-H2XA | 120.0 | C3Y-C2Y-H2YA | 120.0 |
| C4X-C3X-C2X | 119.1 (5) | C4Y-C3Y-C2Y | 120.0 |
| C4X-C3X-C22X | 117.6 (6) | C4Y-C3Y-C22Y | 113.2 (11) |
| C2X-C3X-C22X | 123.3 (5) | C2Y-C3Y-C22Y | 126.3 (11) |
| C3X-C4X-C5X | 120.7 (5) | C3Y-C4Y-C5Y | 120.0 |
| C3X-C4X-H4XA | 119.7 | C3Y-C4Y-H4YA | 120.0 |
| C5X-C4X-H4XA | 119.7 | C5Y-C4Y-H4YA | 120.0 |
| C4X-C5X-C6X | 121.3 (5) | C6Y-C5Y-C4Y | 120.0 |
| C4X-C5X-H5XA | 119.3 | C6Y-C5Y-H5YA | 120.0 |
| C6X-C5X-H5XA | 119.3 | C4Y-C5Y-H5YA | 120.0 |
| C1X-C6X-C5X | 118.0 (4) | C5Y-C6Y-C1Y | 120.0 |
| C1X-C6X-C7X | 122.8 (5) | C5Y-C6Y-C7Y | 119.4 (10) |
| C5X-C6X-C7X | 119.1 (4) | C1Y-C6Y-C7Y | 120.6 (10) |
| C6X-C7X-C12X | 112.4 (4) | C6Y-C7Y-C12Y | 111.6 (10) |
| C6X-C7X-C8X | 110.5 (4) | C6Y-C7Y-C8Y | 110.4 (11) |
| C12X-C7X-C8X | 114.7 (4) | C12Y-C7Y-C8Y | 113.1 (11) |
| C6X-C7X-H7XA | 106.2 | C6Y-C7Y-H7YA | 107.1 |
| C12X-C7X-H7XA | 106.2 | C12Y-C7Y-H7YA | 107.1 |
| C8X-C7X-H7XA | 106.2 | C8Y-C7Y-H7YA | 107.1 |
| C13X-C8X-C15X | 114.1 (4) | C13Y-C8Y-C15Y | 113.6 (10) |
| C13X-C8X-C7X | 112.0 (4) | C13Y-C8Y-C7Y | 110.7 (15) |
| C15X-C8X-C7X | 109.2 (4) | C15Y-C8Y-C7Y | 107.3 (12) |
| C13X-C8X-C9X | 110.2 (4) | C13Y-C8Y-C9Y | 111.1 (14) |
| C15X-C8X-C9X | 103.4 (5) | C15Y-C8Y-C9Y | 105.1 (12) |
| C7X-C8X-C9X | 107.5 (4) | C7Y-C8Y-C9Y | 108.9 (9) |
| C16X-C9X-C10X | 113.2 (4) | C10Y-C9Y-C16Y | 114.0 (12) |
| C16X-C9X-C8X | 111.2 (4) | C10Y-C9Y-C8Y | 115.6 (12) |
| C10X-C9X-C8X | 111.7 (4) | C16Y-C9Y-C8Y | 112.2 (11) |
| C16X-C9X-H9XA | 106.7 | C10Y-C9Y-H9YA | 104.5 |
| C10X-C9X-H9XA | 106.7 | C16Y-C9Y-H9YA | 104.5 |
| C8X-C9X-H9XA | 106.7 | C8Y-C9Y-H9YA | 104.5 |
| C11X-C10X-C9X | 113.3 (4) | C11Y-C10Y-C9Y | 112.6 (12) |
| C11X-C10X-H10A | 108.9 | C11Y-C10Y-H10C | 109.1 |
| C9X-C10X-H10A | 108.9 | C9Y-C10Y-H10C | 109.1 |

**Table S3.** *Cont.*

| 3a | | 3b | |
|---|---|---|---|
| C11X-C10X-H10B | 108.9 | C11Y-C10Y-H10D | 109.1 |
| C9X-C10X-H10B | 108.9 | C9Y-C10Y-H10D | 109.1 |
| H10A-C10X-H10B | 107.7 | H10C-C10Y-H10D | 107.8 |
| O4X-C11X-C10X | 121.2 (5) | O4Y-C11Y-C12Y | 122.2 (14) |
| O4X-C11X-C12X | 122.9 (5) | O4Y-C11Y-C10Y | 123.7 (14) |
| C10X-C11X-C12X | 115.5 (4) | C12Y-C11Y-C10Y | 114.1 (12) |
| C11X-C12X-C7X | 113.6 (4) | C11Y-C12Y-C7Y | 112.1 (11) |
| C11X-C12X-H12A | 108.8 | C11Y-C12Y-H12C | 109.2 |
| C7X-C12X-H12A | 108.8 | C7Y-C12Y-H12C | 109.2 |
| C11X-C12X-H12B | 108.8 | C11Y-C12Y-H12D | 109.2 |
| C7X-C12X-H12B | 108.8 | C7Y-C12Y-H12D | 109.2 |
| H12A-C12X-H12B | 107.7 | H12C-C12Y-H12D | 107.9 |
| O1X-C13X-N1X | 119.7 (5) | O1Y-C13Y-N1Y | 117.1 (16) |
| O1X-C13X-C8X | 121.6 (5) | O1Y-C13Y-C8Y | 123.0 (16) |
| N1X-C13X-C8X | 118.7 (5) | N1Y-C13Y-C8Y | 118.1 (14) |
| O2X-C14X-N2X | 121.4 (7) | O2Y-C14Y-N2Y | 121.4 (17) |
| O2X-C14X-N1X | 120.9 (6) | O2Y-C14Y-N1Y | 121.4 (17) |
| N2X-C14X-N1X | 117.7 (5) | N2Y-C14Y-N1Y | 117.2 (12) |
| O3X-C15X-N2X | 121.9 (6) | O3Y-C15Y-N2Y | 121.7 (14) |
| O3X-C15X-C8X | 119.8 (5) | O3Y-C15Y-C8Y | 119.7 (13) |
| N2X-C15X-C8X | 118.2 (5) | N2Y-C15Y-C8Y | 118.5 (13) |
| C21X-C16X-C17X | 117.2 (5) | C17Y-C16Y-C21Y | 120.0 |
| C21X-C16X-C9X | 121.9 (5) | C17Y-C16Y-C9Y | 117.2 (11) |
| C17X-C16X-C9X | 120.9 (5) | C21Y-C16Y-C9Y | 122.7 (11) |
| C18X-C17X-C16X | 122.2 (5) | C16Y-C17Y-C18Y | 120.0 |
| C18X-C17X-H17A | 118.9 | C16Y-C17Y-H17B | 120.0 |
| C16X-C17X-H17A | 118.9 | C18Y-C17Y-H17B | 120.0 |
| C17X-C18X-C19X | 120.1 (5) | C19Y-C18Y-C17Y | 120.0 |
| C17X-C18X-H18A | 119.9 | C19Y-C18Y-H18B | 120.0 |
| C19X-C18X-H18A | 119.9 | C17Y-C18Y-H18B | 120.0 |
| C18X-C19X-C20X | 118.2 (5) | C18Y-C19Y-C20Y | 120.0 |
| C18X-C19X-C25X | 119.1 (5) | C18Y-C19Y-C25Y | 113.5 (12) |
| C20X-C19X-C25X | 122.7 (5) | C20Y-C19Y-C25Y | 126.4 (12) |
| C21X-C20X-C19X | 121.0 (5) | C19Y-C20Y-C21Y | 120.0 |
| C21X-C20X-H20A | 119.5 | C19Y-C20Y-H20B | 120.0 |
| C19X-C20X-H20A | 119.5 | C21Y-C20Y-H20B | 120.0 |
| C20X-C21X-C16X | 121.4 (5) | C20Y-C21Y-C16Y | 120.0 |
| C20X-C21X-H21A | 119.3 | C20Y-C21Y-H21B | 120.0 |
| C16X-C21X-H21A | 119.3 | C16Y-C21Y-H21B | 120.0 |
| F3X-C22X-F1X | 107.2 (6) | F3Y-C22Y-F2Y | 111.5 (17) |
| F3X-C22X-F2X | 106.6 (5) | F3Y-C22Y-F1Y | 113.4 (19) |
| F1X-C22X-F2X | 105.2 (5) | F2Y-C22Y-F1Y | 108.3 (17) |
| F3X-C22X-C3X | 113.9 (5) | F3Y-C22Y-C3Y | 111.5 (17) |
| F1X-C22X-C3X | 111.5 (5) | F2Y-C22Y-C3Y | 108.8 (17) |
| F2X-C22X-C3X | 112.0 (6) | F1Y-C22Y-C3Y | 103.0 (15) |

**Table S3.** *Cont.*

| 3a | | 3b | |
|---|---|---|---|
| N1X-C23X-H23A | 109.5 | N1Y-C23Y-H23D | 109.5 |
| N1X-C23X-H23B | 109.5 | N1Y-C23Y-H23E | 109.5 |
| H23A-C23X-H23B | 109.5 | H23D-C23Y-H23E | 109.5 |
| N1X-C23X-H23C | 109.5 | N1Y-C23Y-H23F | 109.5 |
| H23A-C23X-H23C | 109.5 | H23D-C23Y-H23F | 109.5 |
| H23B-C23X-H23C | 109.5 | H23E-C23Y-H23F | 109.5 |
| N2X-C24X-H24A | 109.5 | N2Y-C24Y-H24E | 109.5 |
| N2X-C24X-H24B | 109.5 | N2Y-C24Y-H24F | 109.5 |
| H24A-C24X-H24B | 109.5 | H24E-C24Y-H24F | 109.5 |
| N2X-C24X-H24C | 109.5 | N2Y-C24Y-H24G | 109.5 |
| H24A-C24X-H24C | 109.5 | H24E-C24Y-H24G | 109.5 |
| H24B-C24X-H24C | 109.5 | H24F-C24Y-H24G | 109.5 |
| F6X-C25X-F4X | 105.8 (6) | F6Y-C25Y-F4Y | 108.4 (16) |
| F6X-C25X-F5X | 103.6 (5) | F6Y-C25Y-F5Y | 105.1 (16) |
| F4X-C25X-F5X | 102.3 (6) | F4Y-C25Y-F5Y | 103.3 (15) |
| F6X-C25X-C19X | 115.9 (6) | F6Y-C25Y-C19Y | 115.3 (16) |
| F4X-C25X-C19X | 115.4 (5) | F4Y-C25Y-C19Y | 111.1 (16) |
| F5X-C25X-C19X | 112.3 (5) | F5Y-C25Y-C19Y | 112.8 (16) |

**Table S4.** Torsion Angles.

| 3a | | 3b | |
|---|---|---|---|
| C6X_a-C1X_a-C2X_a-C3X_a | 1.1 (9) | C6Y-C1Y-C2Y-C3Y | 0.0 |
| C1X-C2X-C3X-C4X | −1.7 (8) | C1Y-C2Y-C3Y-C4Y | 0.0 |
| C1X-C2X-C3X-C22X | 177.5 (7) | C1Y-C2Y-C3Y-C22Y | −171 (2) |
| C2X-C3X-C4X-C5X | 1.2 (8) | C2Y-C3Y-C4Y-C5Y | 0.0 |
| C22X-C3X-C4X-C5X | −178.0 (7) | C22Y-C3Y-C4Y-C5Y | 172.2 (19) |
| C3X-C4X-C5X-C6X | −0.2 (8) | C3Y-C4Y-C5Y-C6Y | 0.0 |
| C2X-C1X-C6X-C5X | −0.1 (8) | C4Y-C5Y-C6Y-C1Y | 0.0 |
| C2X-C1X-C6X-C7X | −178.2 (6) | C4Y-C5Y-C6Y-C7Y | −179.4 (16) |
| C4X-C5X-C6X-C1X | −0.3 (7) | C2Y-C1Y-C6Y-C5Y | 0.0 |
| C4X-C5X-C6X-C7X | 177.8 (5) | C2Y-C1Y-C6Y-C7Y | 179.4 (17) |
| C1X-C6X-C7X-C12X | −42.6 (7) | C5Y-C6Y-C7Y-C12Y | 153.7 (13) |
| C5X-C6X-C7X-C12X | 139.4 (5) | C1Y-C6Y-C7Y-C12Y | −25.7 (18) |
| C1X-C6X-C7X-C8X | 86.9 (6) | C5Y-C6Y-C7Y-C8Y | −79.6 (16) |
| C5X-C6X-C7X-C8X | −91.2 (6) | C1Y-C6Y-C7Y-C8Y | 101.1 (14) |
| C6X-C7X-C8X-C13X | −59.8 (5) | C6Y-C7Y-C8Y-C13Y | −53.7 (16) |
| C12X-C7X-C8X-C13X | 68.5 (5) | C12Y-C7Y-C8Y-C13Y | 72.2 (15) |
| C6X-C7X-C8X-C15X | 67.6 (5) | C6Y-C7Y-C8Y-C15Y | 70.8 (15) |
| C12X-C7X-C8X-C15X | −164.2 (5) | C12Y-C7Y-C8Y-C15Y | −163.3 (13) |
| C6X-C7X-C8X-C9X | 179.1 (4) | C6Y-C7Y-C8Y-C9Y | −176.0 (13) |
| C12X-C7X-C8X-C9X | −52.6 (5) | C12Y-C7Y-C8Y-C9Y | −50.1 (16) |
| C13X-C8X-C9X-C16X | 60.9 (6) | C13Y-C8Y-C9Y-C10Y | −73.5 (17) |
| C15X-C8X-C9X-C16X | −61.4 (5) | C15Y-C8Y-C9Y-C10Y | 163.2 (15) |
| C7X-C8X-C9X-C16X | −176.8 (4) | C7Y-C8Y-C9Y-C10Y | 48.6 (17) |
| C13X-C8X-C9X-C10X | −66.6 (6) | C13Y-C8Y-C9Y-C16Y | 59.5 (18) |
| C15X-C8X-C9X-C10X | 171.0 (5) | C15Y-C8Y-C9Y-C16Y | −63.8 (16) |
| C7X-C8X-C9X-C10X | 55.6 (5) | C7Y-C8Y-C9Y-C16Y | −178.4 (13) |
| C16X-C9X-C10X-C11X | 178.7 (4) | C16Y-C9Y-C10Y-C11Y | 178.7 (15) |
| C8X-C9X-C10X-C11X | −54.8 (6) | C8Y-C9Y-C10Y-C11Y | −49 (2) |
| C9X-C10X-C11X-O4X | −138.4 (6) | C9Y-C10Y-C11Y-O4Y | −131 (2) |
| C9X-C10X-C11X-C12X | 48.6 (7) | C9Y-C10Y-C11Y-C12Y | 51 (2) |
| O4X-C11X-C12X-C7X | 142.9 (6) | O4Y-C11Y-C12Y-C7Y | 128 (2) |
| C10X-C11X-C12X-C7X | −44.3 (7) | C10Y-C11Y-C12Y-C7Y | −53 (2) |
| C6X-C7X-C12X-C11X | 175.3 (4) | C6Y-C7Y-C12Y-C11Y | 179.2 (14) |
| C8X-C7X-C12X-C11X | 48.0 (6) | C8Y-C7Y-C12Y-C11Y | 53.9 (18) |
| C14X-N1X-C13X-O1X | 176.2 (7) | C14Y-N1Y-C13Y-O1Y | −180 (7) |
| C23X-N1X-C13X-O1X | −5.7 (8) | C23Y-N1Y-C13Y-O1Y | −13 (10) |
| C14X-N1X-C13X-C8X | −6.1 (9) | C14Y-N1Y-C13Y-C8Y | 15 (11) |
| C23X-N1X-C13X-C8X | 172.0 (5) | C23Y-N1Y-C13Y-C8Y | −179 (6) |
| C15X-C8X-C13X-O1X | −171.9 (5) | C15Y-C8Y-C13Y-O1Y | −180 (3) |
| C7X-C8X-C13X-O1X | −47.3 (7) | C7Y-C8Y-C13Y-O1Y | −59 (3) |
| C9X-C8X-C13X-O1X | 72.2 (6) | C9Y-C8Y-C13Y-O1Y | 62 (3) |
| C15X-C8X-C13X-N1X | 10.4 (8) | C15Y-C8Y-C13Y-N1Y | −15 (6) |
| C7X-C8X-C13X-N1X | 135.0 (5) | C7Y-C8Y-C13Y-N1Y | 106 (5) |
| C9X-C8X-C13X-N1X | −105.5 (5) | C9Y-C8Y-C13Y-N1Y | −133 (5) |
| C15X-N2X-C14X-O2X | −177.2 (16) | C15Y-N2Y-C14Y-O2Y | 180 (4) |
| C24X-N2X-C14X-O2X | −6 (3) | C24Y-N2Y-C14Y-O2Y | 6 (6) |

**Table S4**. *Cont.*

| 3a | | 3b | |
|---|---|---|---|
| C15X-N2X-C14X-N1X | 3 (3) | C15Y-N2Y-C14Y-N1Y | −2 (8) |
| C24X-N2X-C14X-N1X | 174.6 (13) | C24Y-N2Y-C14Y-N1Y | −175 (6) |
| C13X-N1X-C14X-O2X | 179.4 (8) | C13Y-N1Y-C14Y-O2Y | 172 (7) |
| C23X-N1X-C14X-O2X | 1.3 (12) | C23Y-N1Y-C14Y-O2Y | 6 (11) |
| C13X-N1X-C14X-N2X | −1.0 (17) | C13Y-N1Y-C14Y-N2Y | −6 (12) |
| C23X-N1X-C14X-N2X | −179.1 (14) | C23Y-N1Y-C14Y-N2Y | −172 (6) |
| C14X-N2X-C15X-O3X | −179.7 (16) | C14Y-N2Y-C15Y-O3Y | −175 (4) |
| C24X-N2X-C15X-O3X | 9 (2) | C24Y-N2Y-C15Y-O3Y | −2 (4) |
| C14X-N2X-C15X-C8X | 2 (3) | C14Y-N2Y-C15Y-C8Y | 0 (5) |
| C24X-N2X-C15X-C8X | −169.5 (12) | C24Y-N2Y-C15Y-C8Y | 173 (2) |
| C13X-C8X-C15X-O3X | 173.2 (6) | C13Y-C8Y-C15Y-O3Y | −176 (2) |
| C7X-C8X-C15X-O3X | 47.1 (8) | C7Y-C8Y-C15Y-O3Y | 61 (2) |
| C9X-C8X-C15X-O3X | −67.1 (8) | C9Y-C8Y-C15Y-O3Y | −54 (2) |
| C13X-C8X-C15X-N2X | −8.5 (14) | C13Y-C8Y-C15Y-N2Y | 8 (3) |
| C7X-C8X-C15X-N2X | −134.6 (13) | C7Y-C8Y-C15Y-N2Y | −114 (2) |
| C9X-C8X-C15X-N2X | 111.2 (13) | C9Y-C8Y-C15Y-N2Y | 130 (2) |
| C10X-C9X-C16X-C21X | 50.5 (7) | C10Y-C9Y-C16Y-C17Y | −125.9 (17) |
| C8X-C9X-C16X-C21X | −76.3 (6) | C8Y-C9Y-C16Y-C17Y | 100.3 (16) |
| C10X-C9X-C16X-C17X | −130.8 (5) | C10Y-C9Y-C16Y-C21Y | 57 (2) |
| C8X-C9X-C16X-C17X | 102.4 (6) | C8Y-C9Y-C16Y-C21Y | −77.2 (18) |
| C21X-C16X-C17X-C18X | 1.0 (8) | C21Y-C16Y-C17Y-C18Y | 0.0 |
| C9X-C16X-C17X-C18X | −177.8 (5) | C9Y-C16Y-C17Y-C18Y | −177.6 (17) |
| C16X-C17X-C18X-C19X | −0.5 (9) | C16Y-C17Y-C18Y-C19Y | 0.0 |
| C17X-C18X-C19X-C20X | −0.4 (8) | C17Y-C18Y-C19Y-C20Y | 0.0 |
| C17X-C18X-C19X-C25X | −179.6 (6) | C17Y-C18Y-C19Y-C25Y | 176 (2) |
| C18X-C19X-C20X-C21X | 0.8 (8) | C18Y-C19Y-C20Y-C21Y | 0.0 |
| C25X-C19X-C20X-C21X | 180.0 (6) | C25Y-C19Y-C20Y-C21Y | −175 (3) |
| C19X-C20X-C21X-C16X | −0.3 (9) | C19Y-C20Y-C21Y-C16Y | 0.0 |
| C17X-C16X-C21X-C20X | −0.6 (8) | C17Y-C16Y-C21Y-C20Y | 0.0 |
| C9X-C16X-C21X-C20X | 178.2 (5) | C9Y-C16Y-C21Y-C20Y | 177.5 (18) |
| C4X-C3X-C22X-F3X | 73.4 (7) | C4Y-C3Y-C22Y-F3Y | 141.2 (17) |
| C2X-C3X-C22X-F3X | −105.8 (7) | C2Y-C3Y-C22Y-F3Y | −47 (2) |
| C4X-C3X-C22X-F1X | −48.0 (8) | C4Y-C3Y-C22Y-F2Y | −95.4 (18) |
| C2X-C3X-C22X-F1X | 132.8 (7) | C2Y-C3Y-C22Y-F2Y | 76 (2) |
| C4X-C3X-C22X-F2X | −165.6 (6) | C4Y-C3Y-C22Y-F1Y | 19 (2) |
| C2X-C3X-C22X-F2X | 15.2 (9) | C2Y-C3Y-C22Y-F1Y | −169.0 (15) |
| C18X-C19X-C25X-F6X | −176.7 (6) | C18Y-C19Y-C25Y-F6Y | 165.6 (17) |
| C20X-C19X-C25X-F6X | 4.2 (9) | C20Y-C19Y-C25Y-F6Y | −19 (3) |
| C18X-C19X-C25X-F4X | −52.3 (8) | C18Y-C19Y-C25Y-F4Y | −71 (2) |
| C20X-C19X-C25X-F4X | 128.6 (7) | C20Y-C19Y-C25Y-F4Y | 105 (2) |
| C18X-C19X-C25X-F5X | 64.4 (7) | C18Y-C19Y-C25Y-F5Y | 45 (2) |
| C20X-C19X-C25X-F5X | −114.7 (7) | C20Y-C19Y-C25Y-F5Y | −139.8 (18) |

All esds (except the esd in the dihedral angle between two l.s. planes) are estimated using the full covariance matrix. The cell esds are taken into account individually in the estimation of esds in distances, angles and torsion angles; correlations between esds in cell parameters are only used when they are defined by crystal symmetry. An approximate (isotropic) treatment of cell esds is used for estimating esds involving l.s. planes.

**Table S5.** Hydrogen-bond geometry (Å, °) of **3a**.

| ***D*-H···*A*** | ***D*-H** | **H···*A*** | ***D*···*A*** | ***D*-H···*A*** |
|---|---|---|---|---|
| C2-H2A···O3$^{i}$ | 0.9300 | 2.5700 | 3.2005 (17) | 125.00 |
| C9-H9A···O2$^{ii}$ | 0.9800 | 2.4900 | 3.2986 (17) | 139.00 |
| C21-H21A···O1 | 0.9300 | 2.6000 | 3.2311 (18) | 126.00 |
| C24-H24B···F1$^{iii}$ | 0.9600 | 2.5000 | 3.4021 (19) | 156.00 |
| C24-H24C···O4$^{iv}$ | 0.9600 | 2.5300 | 3.132 (2) | 121.00 |

Symmetry codes: (i) *x*, *y* − 1, *z*; (ii) *x*, −*y*, *z* − 1/2; (iii) –*x* + 1/2, *y* + 1/2, −*z* + 1/2; (iv) *x*, −*y* + 1, *z* + 1/2

**Table S6.** Hydrogen-bond geometry (Å, °) of **3b** (full molecule disorder).

| ***D*-H···*A*** | ***D*-H** | **H···*A*** | ***D*···*A*** | ***D*-H···*A*** |
|---|---|---|---|---|
| C4X-H4XA···F5X$^{i}$ | 0.9300 | 2.4800 | 3.362 (8) | 157.00 |
| C10X-H10B···O3X$^{ii}$ | 0.9700 | 2.2300 | 3.143 (6) | 156.00 |
| C23X-H23A···F4X$^{iii}$ | 0.9600 | 2.5300 | 3.481 (8) | 171.00 |
| C23X-H23B···O2X$^{iii}$ | 0.9600 | 2.4700 | 3.430 (8) | 179.00 |
| C24X-H24A···F3X$^{iv}$ | 0.9600 | 2.5200 | 3.332 (10) | 143.00 |

Symmetry codes: (i) *y*, −*x* + 2, −*z* + 1; (ii) –*y* + 2, *x*, −*z* + 1; (iii) –*y* + 3/2, −*x* + 3/2, *z* + 1/2; (iv) –*y* + 3/2, −*x* + 3/2, *z* − 1/2.

**Table S7.** The calculated and experimental geometric parameters of the studied compounds using B3LYP/6-311G(d,p) method.

| Parameter [a] | 3a | | | | | Parameter [a] | 3b | | | | |
|---|---|---|---|---|---|---|---|---|---|---|---|
| | Calc. | Exp | Parameter [a] | Calc. | Exp | | Calc. | Exp | Parameter [a] | Calc. | Exp |
| R(1-13) | 1.350 | 1.360 | A(1-13-11) | 119.2 | 118.3 | R(1-30) | 1.354 | 1.343 | A(4-47-36) | 121.1 | 120.9 |
| R(2-39) | 1.350 | 1.366 | A(1-13-14) | 119.1 | 118.4 | R(2-30) | 1.352 | 1.344 | A(5-48-34) | 121.5 | 121.9 |
| R(3-31) | 1.215 | 1.217 | A(2-39-37) | 119.1 | 118.7 | R(3-30) | 1.349 | 1.311 | A(5-48-45) | 120.0 | 119.8 |
| R(4-32) | 1.209 | 1.210 | A(2-39-40) | 119.2 | 118.5 | R(4-21) | 1.384 | 1.384 | A(6-18-15) | 122.4 | 122.9 |
| R(5-33) | 1.219 | 1.218 | A(3-31-7) | 121.3 | 120.4 | R(4-47) | 1.399 | 1.385 | A(6-18-19) | 122.4 | 121.2 |
| R(6-27) | 1.210 | 1.210 | A(3-31-21) | 120.1 | 120.9 | R(4-51) | 1.473 | 1.468 | A(9-7-14) | 121.2 | 122.2 |
| R(7-31) | 1.384 | 1.377 | A(4-32-7) | 120.8 | 121.1 | R(5-48) | 1.215 | 1.210 | A(7-9-49) | 119.8 | 120.1 |
| R(7-32) | 1.396 | 1.390 | A(4-32-8) | 122.1 | 121.7 | R(6-18) | 1.209 | 1.200 | A(7-14-12) | 118.3 | 117.2 |
| R(7-44) | 1.473 | 1.469 | A(5-33-8) | 119.6 | 120.2 | R(7-9) | 1.391 | 1.379 | A(7-14-46) | 119.5 | 120.9 |
| R(8-32) | 1.401 | 1.386 | A(5-33-21) | 121.6 | 120.7 | R(7-14) | 1.400 | 1.387 | A(9-49-11) | 119.8 | 118.2 |
| R(8-33) | 1.382 | 1.379 | A(6-27-24) | 122.4 | 121.8 | R(9-49) | 1.393 | 1.383 | A(9-49-55) | 120.3 | 119.1 |
| R(8-48) | 1.472 | 1.471 | A(6-27-28) | 122.4 | 121.3 | R(11-12) | 1.390 | 1.377 | A(12-11-49) | 120.1 | 121.0 |
| R(9-11) | 1.392 | 1.384 | A(31-7-32) | 125.4 | 125.3 | R(11-49) | 1.394 | 1.390 | A(11-12-14) | 120.9 | 121.4 |
| R(9-18) | 1.401 | 1.394 | A(31-7-44) | 119.1 | 118.1 | R(12-14) | 1.401 | 1.381 | A(11-49-55) | 119.8 | 122.7 |
| R(11-13) | 1.385 | 1.376 | A(7-31-21) | 118.6 | 118.7 | R(14-46) | 1.524 | 1.525 | A(12-14-46) | 122.2 | 121.9 |
| R(13-14) | 1.386 | 1.376 | A(32-7-44) | 115.5 | 116.6 | R(15-18) | 1.518 | 1.498 | A(14-46-19) | 112.5 | 113.2 |
| R(14-16) | 1.392 | 1.394 | A(7-32-8) | 117.1 | 117.2 | R(15-43) | 1.542 | 1.543 | A(14-46-45) | 113.1 | 111.2 |
| R(16-18) | 1.402 | 1.397 | A(32-8-33) | 125.3 | 125.0 | R(18-19) | 1.518 | 1.495 | A(18-15-43) | 112.5 | 113.6 |
| R(18-19) | 1.524 | 1.521 | A(32-8-48) | 117.7 | 117.3 | R(19-46) | 1.542 | 1.529 | A(15-18-19) | 115.2 | 115.5 |
| R(19-21) | 1.586 | 1.580 | A(33-8-48) | 117.0 | 117.6 | R(21-35) | 1.219 | 1.223 | A(15-43-42) | 112.5 | 112.4 |
| R(19-28) | 1.542 | 1.531 | A(8-33-21) | 118.8 | 119.0 | R(21-45) | 1.524 | 1.510 | A(15-43-45) | 113.4 | 114.7 |

**Table S7.** *Cont.*

| Parameter [a] | 3a | | | | | Parameter [a] | 3b | | | | |
|---|---|---|---|---|---|---|---|---|---|---|---|
| | Calc. | Exp | Parameter [a] | Calc. | Exp | | Calc. | Exp | Parameter [a] | Calc. | Exp |
| R(21-22) | 1.586 | 1.567 | A(11-9-18) | 121.4 | 121.2 | R(22-24) | 1.389 | 1.403 | A(18-19-46) | 112.5 | 113.3 |
| R(21-31) | 1.534 | 1.519 | A(9-11-13) | 118.6 | 118.2 | R(22-42) | 1.402 | 1.381 | A(19-46-45) | 113.4 | 111.7 |
| R(21-33) | 1.521 | 1.513 | A(9-18-16) | 118.1 | 118.5 | R(24-37) | 1.396 | 1.394 | A(35-21-45) | 120.8 | 121.6 |
| R(22-24) | 1.542 | 1.533 | A(9-18-19) | 119.5 | 119.1 | R(26-34) | 1.473 | 1.499 | A(21-45-43) | 110.5 | 112.0 |
| R(22-34) | 1.524 | 1.526 | A(11-13-14) | 121.8 | 123.2 | R(30-37) | 1.504 | 1.474 | A(21-45-46) | 110.6 | 110.2 |
| R(24-27) | 1.517 | 1.506 | A(13-14-16) | 118.8 | 117.5 | R(31-55) | 1.354 | 1.346 | A(21-45-48) | 115.2 | 114.1 |
| R(27-28) | 1.517 | 1.508 | A(14-16-18) | 121.2 | 121.3 | R(32-55) | 1.350 | 1.371 | A(24-22-42) | 120.9 | 120.9 |
| R(34-35) | 1.402 | 1.393 | A(16-18-19) | 122.3 | 122.4 | R(33-55) | 1.351 | 1.340 | A(22-24-37) | 120.1 | 120.0 |
| R(34-42) | 1.401 | 1.392 | A(18-19-21) | 113.2 | 111.3 | R(34-47) | 1.396 | 1.350 | A(22-42-40) | 118.3 | 118.0 |
| R(35-37) | 1.392 | 1.391 | A(18-19-28) | 112.7 | 112.6 | R(34-48) | 1.382 | 1.377 | A(22-42-43) | 122.2 | 122.8 |
| R(37-39) | 1.386 | 1.371 | A(21-19-28) | 113.1 | 112.7 | R(36-47) | 1.208 | 1.217 | A(24-37-30) | 119.1 | 123.3 |
| R(39-40) | 1.385 | 1.374 | A(19-21-22) | 108.7 | 108.8 | R(37-38) | 1.392 | 1.374 | A(24-37-38) | 119.8 | 119.1 |
| R(40-42) | 1.392 | 1.394 | A(19-21-31) | 106.0 | 104.4 | R(38-40) | 1.393 | 1.382 | A(26-34-47) | 115.6 | 117.2 |
| | | | A(19-21-33) | 110.6 | 109.9 | R(40-42) | 1.399 | 1.396 | A(26-34-48) | 119.3 | 116.2 |
| | | | A(19-28-27) | 112.5 | 113.5 | R(42-43) | 1.524 | 1.532 | A(30-37-38) | 121.1 | 117.6 |
| | | | A(22-21-31) | 106.0 | 107.6 | R(43-45) | 1.586 | 1.552 | A(31-55-32) | 106.8 | 102.3 |
| | | | A(22-21-33) | 110.6 | 111.4 | R(45-46) | 1.586 | 1.590 | A(31-55-33) | 106.6 | 105.8 |
| | | | A(21-22-24) | 113.1 | 112.8 | R(45-48) | 1.536 | 1.539 | A(31-55-49) | 111.6 | 115.4 |
| | | | A(21-22-34) | 113.2 | 112.3 | R(49-55) | 1.504 | 1.489 | A(32-55-33) | 107.4 | 103.6 |
| | | | A(31-21-33) | 114.8 | 114.5 | A(1-30-2) | 106.5 | 105.2 | A(32-55-49) | 112.2 | 112.3 |
| | | | A(24-22-34) | 112.7 | 112.4 | A(1-30-3) | 107.0 | 107.2 | A(33-55-49) | 112.0 | 115.9 |
| | | | A(22-24-27) | 112.5 | 114.3 | A(1-30-37) | 111.6 | 111.5 | A(47-34-48) | 125.0 | 126.1 |
| | | | A(22-34-35) | 122.3 | 122.6 | A(2-30-3) | 107.3 | 106.6 | A(34-47-36) | 121.3 | 121.4 |
| | | | A(22-34-42) | 119.5 | 119.2 | A(2-30-37) | 111.7 | 112.0 | A(34-48-45) | 118.5 | 118.2 |
| | | | A(24-27-28) | 115.2 | 116.8 | A(3-30-37) | 112.5 | 113.9 | A(37-38-40) | 119.8 | 120.7 |
| | | | A(35-34-42) | 118.1 | 118.1 | A(21-4-47) | 125.3 | 124.3 | A(38-40-42) | 121.2 | 121.3 |
| | | | A(34-35-37) | 121.2 | 121.0 | A(21-4-51) | 119.3 | 118.5 | A(40-42-43) | 119.5 | 119.1 |
| | | | A(34-42-40) | 121.4 | 121.7 | A(4-21-35) | 120.8 | 119.7 | A(42-43-45) | 113.1 | 110.5 |
| | | | A(35-37-39) | 118.8 | 118.6 | A(4-21-45) | 118.3 | 118.7 | A(43-45-46) | 108.3 | 107.5 |
| | | | A(37-39-40) | 121.8 | 122.8 | A(47-4-51) | 115.3 | 117.1 | A(43-45-48) | 105.9 | 109.2 |
| | | | A(39-40-42) | 118.6 | 117.8 | A(4-47-34) | 117.6 | 117.7 | A(46-45-48) | 105.9 | 103.4 |

[a] Atom numbering referred to Figure 5.

**Table S8.** The calculated electronic transitions using TD-DFT method.

| $\lambda_{max}$ (nm) | f | Major Contributions |
|---|---|---|
| | | **3a** |
| 293.2 | 0.0030 | H→L + 2 (45%), H→L + 5 (26%) |
| 282.2 | 0.0008 | H→L (99%) |
| 266.3 | 0.0001 | H-5→L (61%), H-4→L (31%) |
| 258.6 | 0.0001 | H-1→L (98%) |
| 255.7 | 0.0009 | H-2→L (97%) |
| 248.1 | 0.0077 | H→L + 1 (87%) |
| 242.0 | 0.0000 | H-2→L + 1 (11%), H→L + 2 (13%), H→L + 3 (50%) |
| 239.2 | 0.0087 | H-7→L (49%), H-5→L + 1 (18%) |
| 235.3 | 0.0005 | H-3→L (91%) |
| 234.0 | 0.0001 | H-5→L (32%), H-4→L (65%) |
| 233.2 | 0.0064 | H-1→L + 1 (50%) |
| 229.8 | 0.0002 | H-2→L + 1 (32%), H-1→L + 3 (18%), H→L + 2 (14%), H→L + 3 (13%) |
| 228.3 | 0.1530 | H-1→L + 2 (20%), H→L + 2 (22%), H→L + 3 (25%), H→L + 5 (19%) |
| 224.3 | 0.0110 | H-6→L (48%), H-1→L + 1 (17%) |
| 222.5 | 0.0149 | H-2→L + 1 (15%), H-1→L + 2 (52%), H→L + 5 (28%) |
| 222.1 | 0.0169 | H-6→L (42%), H-2→L + 2 (17%), H-1→L + 1 (15%), H→L + 4 (11%) |
| 219.0 | 0.0151 | H-2→L + 1 (30%), H-1→L + 3 (52%) |
| 217.8 | 0.0235 | H-2→L + 2 (49%), H→L + 4 (27%) |
| 217.0 | 0.0003 | H-2→L + 3 (31%), H→L + 4 (40%) |
| 215.6 | 0.0004 | H-9→L (73%) |
| | | **3b** |
| 296.2 | 0.0056 | H→L + 1 (44%), H→L + 5 (45%) |
| 275.9 | 0.0004 | H→L (99%) |
| 266.2 | 0.0001 | H-5→L (15%), H-4→L (22%), H-2→L (55%) |
| 248.5 | 0.0363 | H→L + 1 (51%), H→L + 5 (35%) |
| 244.4 | 0.0020 | H→L + 2 (65%), H→L + 3 (21%) |
| 241.9 | 0.0036 | H-1→L (70%), H→L + 2 (13%) |
| 240.7 | 0.0156 | H-7→L (21%), H-4→L (15%) |
| 239.7 | 0.0063 | H-1→L (21%), H→L + 2 (16%), H→L + 3 (40%) |
| 239.2 | 0.0015 | H-7→L (13%), H-3→L + 1 (12%), H→L + 4 (23%) |
| 234.2 | 0.0006 | H-3→L (83%) |
| 232.9 | 0.0008 | H-4→L (48%), H-2→L (27%), H→L + 4 (11%) |
| 232.2 | 0.0073 | H-2→L + 1 (16%), H→L + 3 (17%) |
| 232.1 | 0.0005 | H-5→L (64%) |
| 229.3 | 0.0050 | H-3→L + 1 (17%), H-1→L + 4 (13%), H→L + 4 (39%) |
| 225.3 | 0.0016 | H-5→L + 1 (17%), H-4→L + 1 (28%), H-2→L + 1 (47%) |
| 224.2 | 0.0266 | H-6→L (84%) |
| 222.1 | 0.2486 | H-1→L + 1 (81%) |
| 217.0 | 0.0000 | H-9→L (79%) |
| 215.8 | 0.0129 | H-5→L + 1 (36%), H-4→L + 1 (35%) |
| 213.7 | 0.0040 | H→L + 6 (79%) |

**Table S9.** The calculated chemical shifts δ (ppm) of the studied compound using GIAO method.

| Atom | $\delta_{calc}$ (ppm) | $\delta_{exp.}$(ppm) | Atom | $\delta_{calc}$ (ppm) | $\delta_{exp.}$(ppm) |
|---|---|---|---|---|---|
| C9 | 137.96 | 126.96 | C7 | 136.21 | 126.96 |
| C11 | 120.65 | 125.48 | C9 | 131.79 | 125.48 |
| C13 | 172.40 | 168.6 | C11 | 132.27 | 125.48 |
| C14 | 121.17 | 125.87 | C12 | 132.56 | 126.96 |
| C16 | 133.67 | 126.96 | C14 | 150.40 | 149.93 |
| C18 | 142.03 | 139.84 | C15 | 48.47 | 44.17 |
| C19 | 58.58 | 45.44 | C18 | 208.88 | 205.78 |
| C21 | 68.12 | 61.51 | C19 | 48.53 | 45.44 |
| C22 | 58.58 | 45.44 | C21 | 176.56 | 170.91 |
| C24 | 48.84 | 44.17 | C22 | 132.79 | 126.96 |
| C27 | 209.61 | 205.78 | C24 | 133.09 | 125.48 |
| C28 | 48.84 | 44.17 | C26 | 29.71 | 28.21 |
| C31 | 178.19 | 170.91 | C30 | 134.23 | 125.87 |
| C32 | 154.66 | 149.93 | C37 | 138.63 | 139.84 |
| C33 | 177.97 | 170.91 | C38 | 131.15 | 125.48 |
| C34 | 142.03 | 139.84 | C40 | 135.88 | 126.96 |
| C35 | 133.67 | 126.96 | C42 | 150.34 | 149.93 |
| C37 | 121.17 | 125.87 | C43 | 59.14 | 61.51 |
| C39 | 172.40 | 168.6 | C45 | 67.26 | 61.51 |
| C40 | 120.65 | 125.48 | C46 | 59.21 | 61.51 |
| C42 | 137.96 | 126.96 | C47 | 154.96 | 168.6 |
| C44 | 30.03 | 28.21 | C48 | 177.60 | 170.91 |
| C48 | 30.34 | 28.63 | C49 | 138.58 | 139.84 |
| | | | C51 | 29.20 | 28.63 |
| | | | C55 | 134.24 | 125.87 |
| H10 | 7.22 | 7.04 | H8 | 7.34 | 6.75 |
| H12 | 6.95 | 7.01 | H10 | 7.62 | 7.14 |
| H15 | 7.03 | 7.01 | H13 | 7.48 | 6.75 |
| H17 | 7.34 | 7.04 | H16 | 2.33 | 2.7 |
| H20 | 3.83 | 3.64 | H17 | 3.83 | 4.25 |
| H23 | 3.83 | 3.64 | H20 | 3.83 | 4.28 |
| H25 | 3.76 | 3.95 | H23 | 7.50 | 6.75 |
| H26 | 2.28 | 2.55 | H25 | 7.70 | 7.14 |
| H29 | 2.28 | 2.59 | H27 | 2.20 | 3.02 |
| H30 | 3.76 | 3.99 | H28 | 2.22 | 3.02 |
| H36 | 7.34 | 6.94 | H29 | 4.07 | 3.02 |
| H38 | 7.03 | 6.89 | H39 | 7.63 | 7.14 |
| H41 | 6.95 | 6.89 | H41 | 7.32 | 6.75 |
| H43 | 7.21 | 6.94 | H44 | 3.93 | 3.56 |
| H45 | 4.12 | 3.02 | H50 | 7.70 | 7.14 |
| H46 | 2.24 | 2.88 | H52 | 4.39 | 3.04 |
| H47 | 2.25 | 2.88 | H53 | 2.45 | 3.04 |
| H49 | 3.93 | 3.02 | H54 | 2.45 | 3.04 |
| H50 | 2.70 | 2.88 | H56 | 2.32 | 2.74 |
| H51 | 2.70 | 2.88 | H57 | 3.92 | 3.56 |

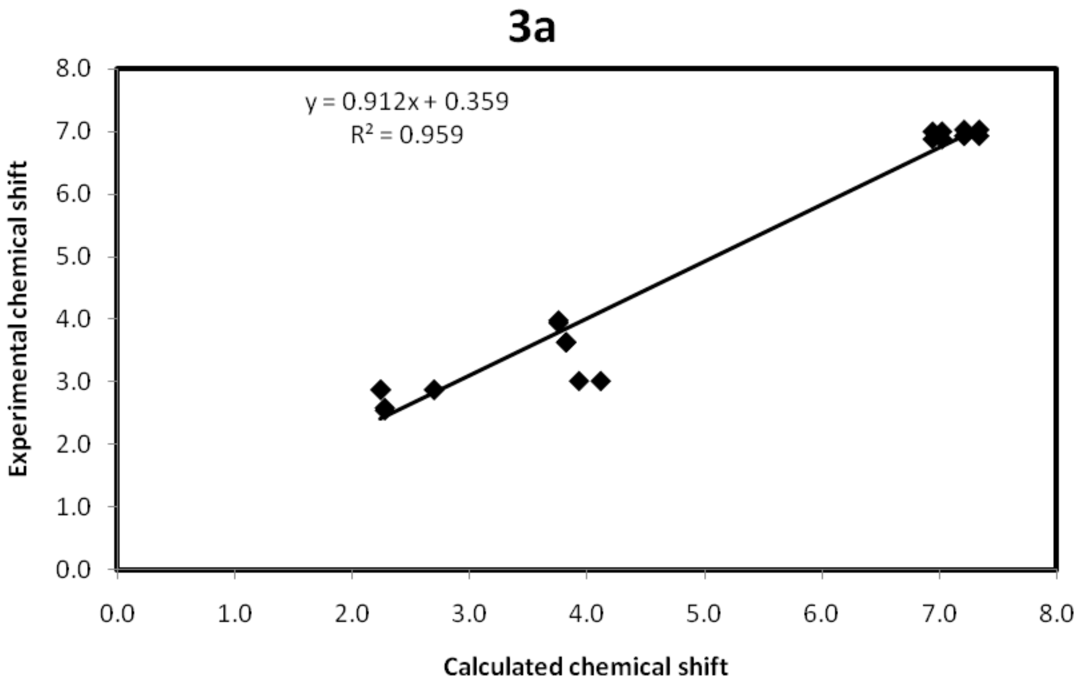


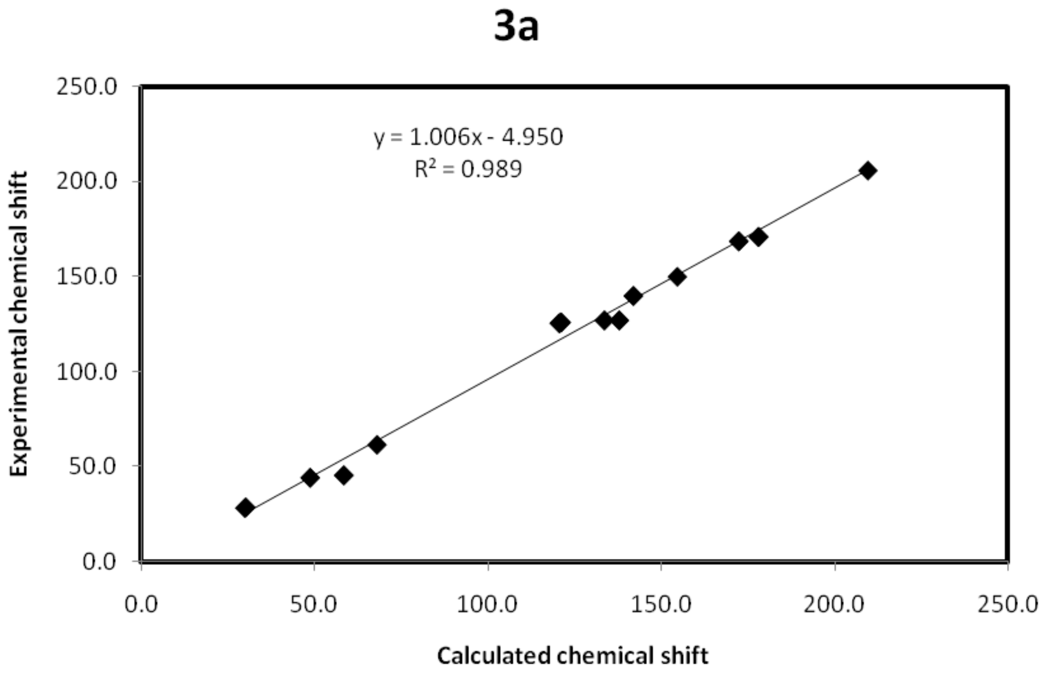


**Figure S7.** The correlation graphs between the calculated and experimental $^{1}$H- and $^{13}$C-NMR chemical shifts of **3a**.

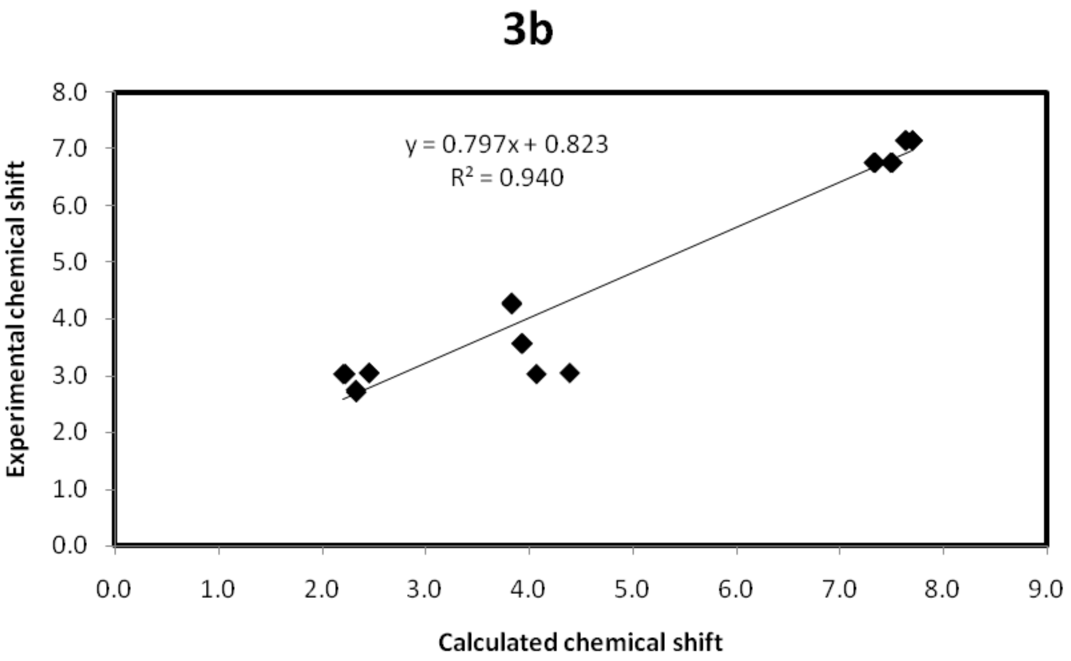


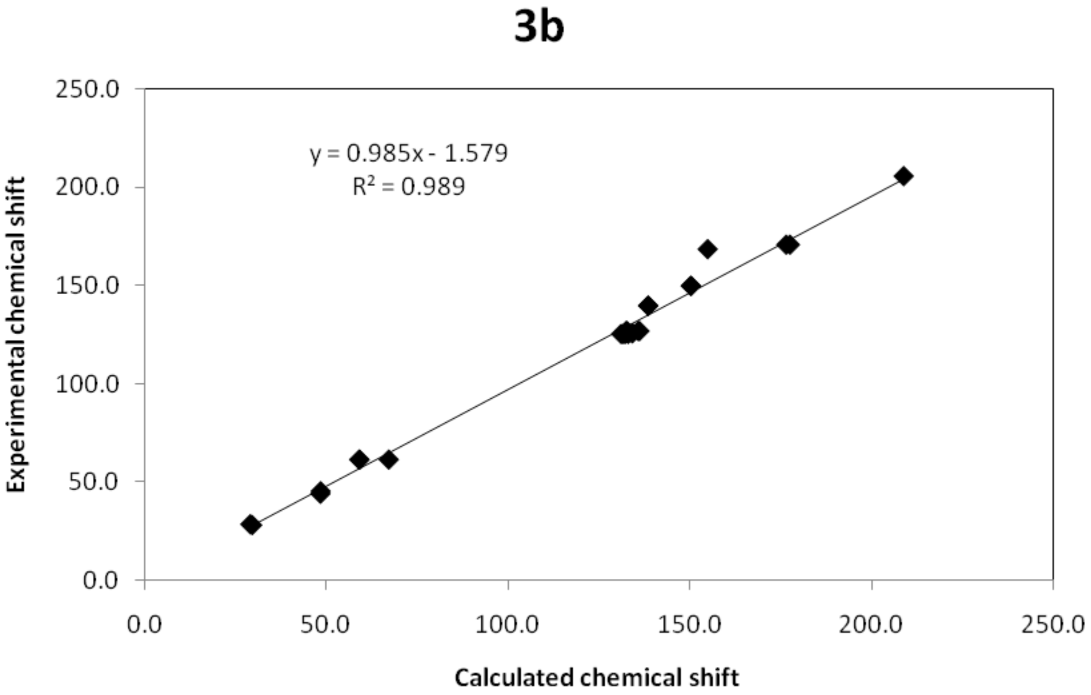


**Figure S8.** The correlation graphs between the calculated and experimental $^{1}$H- and $^{13}$C-NMR chemical shifts of **3b**.
